# Evaluation of Telehealth Services that are Clinically Appropriate for Reimbursement in the US Medicaid Population: Mixed Methods Study

**DOI:** 10.2196/46412

**Published:** 2024-03-28

**Authors:** Sanjeev Saravanakumar, Andrey Ostrovsky

**Affiliations:** 1 GW School of Medicine & Health Sciences Washington, DC United States; 2 Social Innovation Ventures Washington, DC United States

**Keywords:** mobile phone, telehealth, Medicaid reimbursement, health equity, Center for Medicare & Medicaid Services, telemedicine, reimbursement, digital health, Medicaid, Public Health Emergency, access, equity, health insurance, coverage, reimburse, equitable, health policy, telehealth expansion

## Abstract

**Background:**

When the US Department of Health and Human Services instituted a State of Public Health Emergency (PHE) during the COVID-19 pandemic, many telehealth flexibilities were fast-tracked to allow state Medicaid agencies to reimburse new specialty services, sites of care, and mediums such as FaceTime to communicate with patients.. This resulted in expanded access to care for financially vulnerable Medicaid patients, as evidenced by an uptick in telehealth use. Research has mostly focused on telehealth reimbursement for limited use cases such as rural primary care, without broader consideration for how telehealth can be appropriately mainstreamed and maintained.

**Objective:**

This study sought to (1) evaluate the continuation of flexible telehealth reimbursement broadly, beyond the COVID-19 pandemic; (2) analyze the clinical effectiveness of the new telehealth services; and (3) offer code-by-code reimbursement guidance to state Medicaid leaders.

**Methods:**

We surveyed 10 state Medicaid medical directors (MMDs) who are responsible for the scientific and clinical appropriateness of Medicaid policies in their respective states. Participants were asked to complete an internet-based survey with a list of medical billing codes, grouped by service type, and asked if they believed they should be reimbursed by Medicaid on a permanent basis. Additional questions covered more detailed recommendations, such as reimbursing video with audio versus audio-only, guardrails for certain specialty services, and motivations behind responses.

**Results:**

The MMDs felt that the majority of services should be reimbursed via some modality of telehealth after the PHE, with the most support for video combined with audio compared to audio-only. There were exceptions on both ends of the spectrum, where services such as pulmonary diagnostics were not recommended to be reimbursed in any form and services such as psychotherapy for mental health had the most support for audio-only. The vast majority of MMDs were supportive of reimbursement for remote monitoring services, but some preferred to have some reimbursement guardrails. We found that 90% (n=9) of MMDs were supportive of reimbursement for telehealth interprofessional services, while half (n=5) of the respondents felt that there should be continued guardrails for reimbursement. Motivations for continuing reimbursement flexibility were largely attributed to improving access to care, improving outcomes, and improving equity among the Medicaid patient population.

**Conclusions:**

There is a strong clinical endorsement to continue the telehealth flexibility enabled by the PHE, primarily for video combined with audio telehealth, with caution against audio-only telehealth in situations where hands-on intervention is necessary for diagnosis or treatment. There is also support for reimbursing remote monitoring services and telehealth interprofessional services, albeit with guardrails. These results are primarily from a perspective of improving access, outcomes, and equity; other state-specific factors such as fiscal impact and technical implementation may need to be taken into account when considering reimbursement decisions on telehealth.

## Introduction

The COVID-19 pandemic created unprecedented disruption to health care systems, particularly to systems serving the most vulnerable populations, such as beneficiaries of the US Medicaid services. Stay-at-home and social distancing orders forced rapid uptake of new technologies for remote patient care, most notably telehealth. Under the state of public health emergency (PHE), state Medicaid programs allowed for additional flexibility for providers to receive reimbursement for telehealth, including allowing the home as an originating site for patients to use telehealth and expanding allowable technological devices to include mainstream smartphone apps such as FaceTime, WhatsApp, and Skype [1,2]. Many of these technological advances were developed before the pandemic but saw a surge in popularity as the public needed creative solutions to work around the pandemic [1]. We now see that telemedicine adoption and use has expanded, which provides new evidence on the impact of telemedicine on cost, quality, and access for those unsure about implementing telehealth in their own practices.

The US Medicaid program was a major beneficiary of the surge in telehealth popularity, being one of the largest insurers with over 85 million people covered in the United States. Medicaid beneficiaries primarily include people whose income is below the federal poverty level, children, pregnant women, older individuals, and individuals with disabilities [3]. While Medicaid is an entitlement program by the federal government, it is largely state-run, with the Social Security Act (SSA) and associated waivers (section 1115, section 1915b, and section 1915c) providing states the flexibility on Medicaid eligibility, covered benefits, and provider payment rates [3]. The flexibility of Medicaid administration has therefore created state-by-state differences in Medicaid’s day-to-day operations, including the implementation and oversight of telemedicine services [4].

The National Medicaid Medical Directors Network (MMDN) is a nonpartisan, nonprofit collective that helps convene Medicaid leaders across all 50 states to discuss differences in state implementation, share best practices, and solve pressing needs among the states [5]. State Medicaid medical directors (MMDs) are typically responsible for ensuring the scientific and clinical appropriateness of state Medicaid policies. When the PHE provided flexibility for telehealth reimbursement during the COVID-19 pandemic, each state was guided by its respective medical director to responsibly ramp up its telehealth capabilities. Historically, these benefits were limited to rural areas or special circumstances for those with intellectual and developmental disabilities [6]. During the COVID-19 pandemic, the expansion of these benefits to all Medicaid beneficiaries marked a period of unprecedented telehealth access.

Before the pandemic, telehealth use represented a negligible amount (<1%) of total outpatient visits, exploded to 13% during the March 2020 to August 2020 period, and has since stabilized to a new level of 8% from March 2021 to August 2021 [1]. There was high variability in telemedicine uptake based on specialty ranging from endocrinologists (67.2%), gastroenterologists (57%), neurologists (56.3%), pain management physicians (50.6%), psychiatrists (50.2%), orthopedic surgeons (20.7%), and ophthalmologists (9.3%) using any telemedicine during the pandemic [7]. While telehealth offers numerous benefits to patients and the Medicaid program, including improved access to care, the impacts on clinical quality and financial maintainability remain unclear [2]. State Medicaid programs are facing budget constraints, forcing conversations about the cost-effectiveness of telehealth services going forward [5]. While numerous questions remain, the growing potential of telehealth has led to several legislative efforts to consider extending telehealth flexibility beyond the PHE and making it permanent [8].

Given the implications of these changes, state Medicaid leaders have expressed needing code-by-code guidance on the specific services that are appropriate to reimburse when delivered via telehealth. Because of the expansion flexibility paired with state-to-state differences in implementation, an existing universal precedent has yet to be established. The 2021 MMDN report and Government Accountability Office (GAO) report on considerations for telehealth reimbursement continuation delve into the topic but do not provide strong prescriptive recommendations.

This paper aims to aggregate the expertise of leadership who oversaw state telehealth expansion during the COVID-19 pandemic, identify code-by-code reimbursement guidance, and provide recommendations at-large for state Medicaid directors. Stakeholders such as providers, payers, and patients are important in shaping this conversation and should be considered with the perspective of the MMDs provided in this study. It is also historically evident that there are challenges for telehealth expansion, which range across cost, technology, and organizational domains, making these data all the more important for the exchange of lessons learned from state to state and to provide guidance for leaders both in the United States and abroad who are uncertain about risk and financial reimbursement policies [9].

We hypothesized that MMDs would advocate to continue reimbursing video and audio for most specialty service codes but would be wary of audio-only telehealth for certain conditions. We summarize and synthesize the findings from a survey of the MMDN which offers evidence-backed input through a lens of clinical appropriateness and quality.

## Methods

### Overview

Study participants were selected from the MMDN, a collaborative platform for senior clinical leaders within state Medicaid agencies, which meets periodically to exchange best practices aimed at enhancing the health outcomes of Medicaid beneficiaries [5]. Considering the study’s objective to discern the clinical justifiability for telehealth reimbursement, engaging MMDs as respondents for this survey was ideal.

The survey was designed to capture MMD’s viewpoints on Medicaid’s reimbursement and benefit coverage policies for telehealth services extending beyond the PHE phase (Multimedia Appendix 1). The formulation of survey questions was informed by an examination of 10 state Medicaid program bulletins—namely, California, Massachusetts, New York, Florida, Pennsylvania, Michigan, North Carolina, Georgia, Ohio, and Illinois—concerning coding and coverage specifications for telehealth services amid the PHE toward the end of 2020 (Multimedia Appendix 2). Prior to distribution, the survey underwent a pilot test conducted by a medical director specializing in virtual care at a major academic medical center, leading to revisions that enhanced the readability and precision of question content. The survey, disseminated via email, was self-administered and required approximately 10-12 minutes for completion. Participants were presented with a compilation of Current Procedural Terminology (CPT) codes and Healthcare Common Procedure Coding System (HCPCS) codes, organized according to service type, and were instructed to indicate which services they deemed warranting permanent Medicaid reimbursement after the PHE. Further inquiries were made regarding the preferred modes of service delivery, encompassing both video and audio, video with audio only, audio-only, or an option for respondents unfamiliar with the code. Regarding a set of services traditionally offered over the telephone, respondents were queried on the appropriateness of categorizing and reimbursing these as telehealth services by Medicaid beyond the PHE, with the home identified as the originating site, and whether such coverage should be extended with or without specific guardrails. The concluding segment of the survey sought to understand the rationale underpinning the respondents’ selections in the preceding sections. For a full examination of the survey, refer to Multimedia Appendix 1.

In collaboration with Academy Health, we distributed the survey to all MMDs (N=50) between January 5, 2021, and February 5, 2021. Qualtrics was the designated survey software and AcademyHealth directly sent the survey via email to all the MMDs. To increase the response rate, a single follow-up email was sent a week later. There was no compensation for completing the survey.

To understand the implications of the survey responses, we used a mixed methods analytical approach. Quantitative data from the survey were analyzed using descriptive statistics to identify trends and patterns in the responses. This included the percentage of respondents who supported the continuation of telehealth reimbursement for specific services, as well as the preferred modality of telehealth (video with audio vs audio-only). We also examined the distribution of responses across different service types and specialties. Qualitative data from the open-ended questions were analyzed thematically to gain insights into the motivations behind the responses and to identify potential guardrails for certain specialty services. This analytical approach allowed us to appreciate the complexities of telehealth reimbursement to provide nuanced recommendations for state Medicaid leaders.

The analysis in our study was primarily descriptive, aimed at discerning the perspectives of MMDs regarding the continued reimbursement for services rendered via telehealth post the PHE.

Given the constraint of small sample sizes, we refrained from performing formal statistical tests that might not yield robust conclusions under such circumstances. To maintain the integrity and relevance of the data, we filtered out responses where less than 50% of the survey questions were completed, ensuring that the analysis was grounded on substantial and meaningful respondent input.

### Ethical Considerations

We did not pursue an institutional review board (IRB) review because our study survey was administered to a nonvulnerable population, entailing no greater than usual risk, with a stringent anonymization process applied to all data collected. This study aligns with the exemption criteria delineated by institutional and regional guidelines, such as those specified by the US Department of Health & Human Services (45 CFR 46.101(b)). Particularly, our methodology resonates with scenarios for which IRB approval may be exempt, including research involving educational tests, surveys, interviews, or observation of public behavior, provided the information obtained is recorded in such a manner that subjects cannot be identified, directly or through identifiers linked to them. This alignment with established guidelines underscores the justification for forgoing IRB approval in our study, thereby adhering to the ethical framework governing human subject research while facilitating timely and resource-efficient data collection. Using diligent data management and confidentiality measures was paramount in our study, particularly as an IRB assessment was not obtained for the survey administered. All survey responses were anonymized at the point of collection, with no personally identifiable information recorded, adhering to the principles of confidentiality and privacy. A robust data management system was used to securely store, process, and analyze the collected data, ensuring restricted access only to authorized members of the research team.

## Results

**Figure 1:**
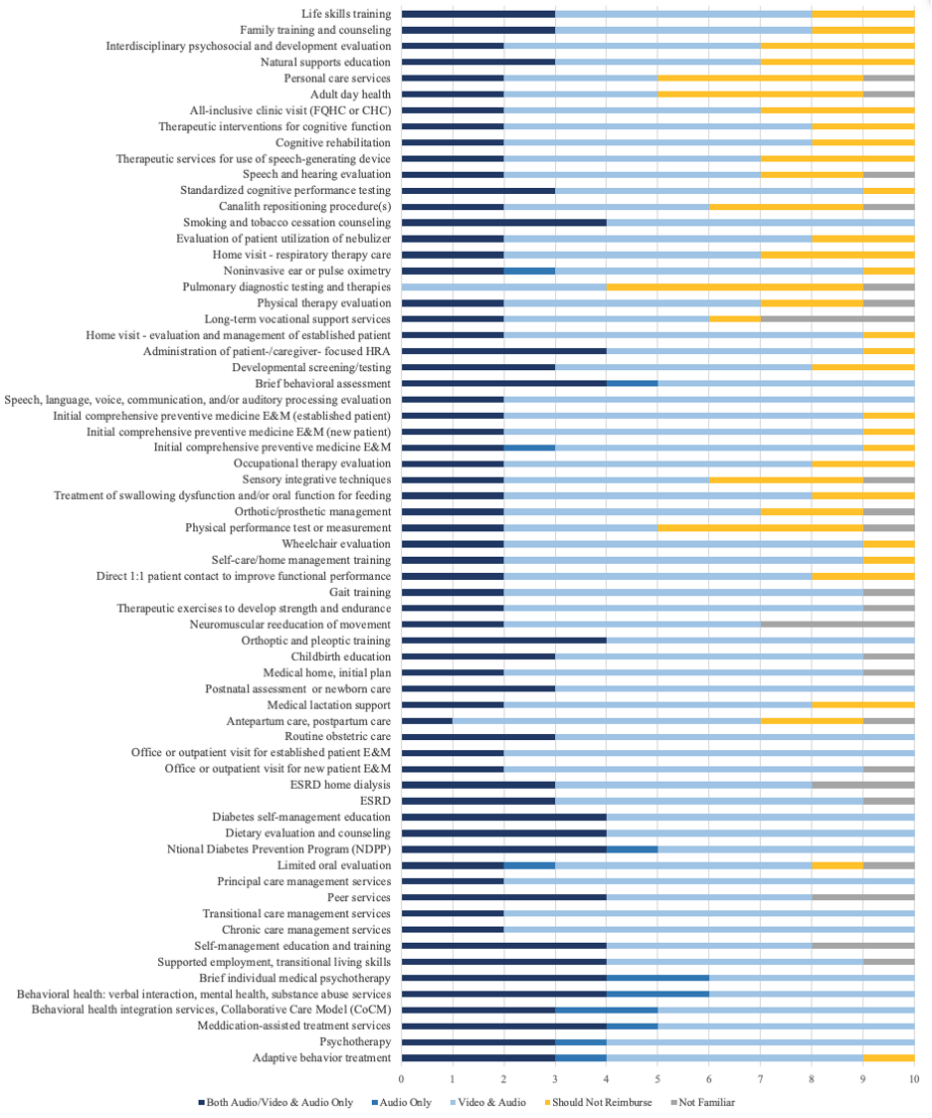
Reimbursement of services that Medicaid beneficiaries can receive via telehealth or telemedicine. CHC: community health center; E&M: evaluation and management; ESRD: end-stage renal disease; FHQC: federally qualified health center; HRA: health risk assessment.

A total of 12 (24%) MMDs completed the survey, and we included 10 respondents in the analysis (20% net response rate), after removing 2 respondents with incomplete surveys. Figure 1 displays the respondents’ preferences for reimbursement for groups of codes that can be received via telehealth or telemedicine. See Multimedia Appendix 2 for specific CPT codes. Respondents felt that the majority of services should be reimbursed via some modality of telehealth after the PHE, though respondents were generally more comfortable with video combined with audio compared to audio-only. The main exception was psychotherapy for mental health or substance use disorder treatment, which had the most support of any services for audio-only with 60% (n=6) of respondents supporting it. There was only one category of codes, pulmonary diagnostics, that a majority of respondents felt should not be reimbursed in any telehealth form; other categories that did not have favorable reimbursement support with 40% (n=4) of respondents including personal care services, adult day services, and physical performance tests.

**Figure 2:**
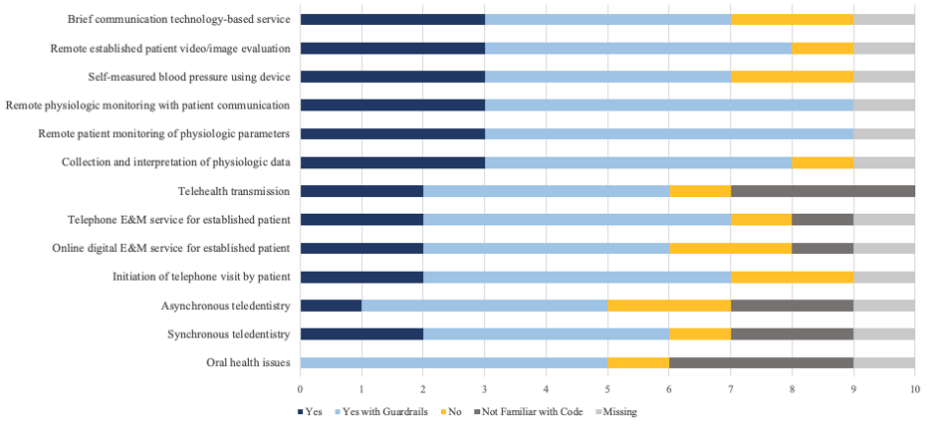
Reimbursement for remote monitoring services and services that Medicaid beneficiaries generally receive by telephone. E&M: evaluation and management.

Figure 2 displays responses about continued reimbursement after the PHE for remote monitoring services and services generally delivered via telephone. Respondents supported continued reimbursement with 70% (n=7) to 90% (n=9) in favor of reimbursing remote monitoring services and 50% (n=5) to 70% (n=7) in favor of reimbursing services that are generally delivered by telephone. However, more than half of supportive respondents felt there should be continued guardrails for reimbursement. The proposed guardrails were inconsistent or not specified across these responses. Responses showed a similar pattern for reimbursement of interprofessional services in Table 1.

When asked about motivations for reimbursement decisions in the previous questions, respondents reported a variety of motivations in Table 2. The most common responses were the service’s likelihood to improve access to care (n=10, 100%), the service’s likelihood to improve outcomes (n=10, 100%), and the service’s likelihood to improve equity (n=8, 80%), while the least common response was the level of evidence for the service and the service’s contribution to the financial sustainability of the Medicaid program (n=3, 30%).

**Table 1:** Telehealth reimbursement of interprofessional services (n=10)^a^.

Responses	Number of MMDs^b^, n
Yes	4
Yes, with guard rails	5
No	1
Not familiar with code	0
Missing response	0

^a^Services include interprofessional telephone or internet or electronic health records assessment and management services provided by a consultative physician, including a verbal and written report to the patient’s treating or requesting physician or other qualified health care professional.

^b^MMD: Medicaid medical director.

**Table 2:** Motivation for telehealth reimbursement recommendation (n=10).

Motivation characteristic	Number of MMDs^a^, n (%)
Service’s likelihood to improve access to care	10 (100)
Service’s likelihood to improve outcomes	10 (100)
Service’s likelihood to improve equity	8 (80)
Service’s likelihood to improve beneficiary experience	7 (70)
Service’s likelihood to reduce provider burden	7 (70)
Service’s cost-effectiveness	7 (70)
Service’s contribution to the financial sustainability of Medicaid program	3 (30)
Level of evidence for the service	3 (30)

^a^MMD: Medicaid medical director.

## Discussion

### Principal Findings

The expansion of telehealth capabilities during the COVID-19 pandemic offered a unique opportunity for clinical leaders to evaluate the strengths and weaknesses of virtually delivered care. The survey administered to MMDs across the United States provides us with insight into the specialty billing codes recommended for reimbursement specifically via telehealth. Several states across the United States were involved in the data aggregation process and the results validated our hypothesis that there would be support for permanent reimbursement for video with audio telehealth after the pandemic for a majority of services with special caution for audio-only telehealth.

Support for continued audio or video telehealth use was prominent; however, there were mixed opinions on continued support for audio-only telehealth from the MMDs. From a clinical perspective, MMDs were hesitant to support audio-only telehealth services where hands-on evaluation or intervention was traditionally necessary. MMDs were concerned about the inability to identify nonverbal cues such as signs of oversedation, anxious fidgeting, or environmental risks such as unsafe housing conditions, which could pose unidentified risks from using telehealth. However, MMDs mostly agree on the usefulness of audio-only telehealth for behavioral health services, aligning with the Centers for Medicare & Medicaid Services (CMS) Physician Fee Schedule 2022 policy approving reimbursement of audio-only telebehavioral health under certain conditions [10].

The benefits of audio-only telehealth are notable, especially in improving access for individuals lacking reliable internet, addressing health equity concerns. Most surveyed MMDs preferred having some regulations if such services are to be reimbursed, to avoid impacting disadvantaged populations adversely. Audio-only services have notably reduced missed appointments and enhanced patient engagement, without compromising care quality in areas like maternal care [11,12].

Furthermore, the results showed support for reimbursing remote monitoring services and telephone-delivered services, though coding and reimbursement challenges have impeded access for Medicaid and Medicare beneficiaries [13]. Ambiguity in coding has historically hindered adoption and patient access to remote physiologic monitoring codes but the creation of new coding options for remote therapeutic monitoring aims to mitigate this issue.

Finally, to address the longstanding issue of specialist availability, MMDs support reimbursing virtual interprofessional services, aiming to attract more specialists to treat Medicaid beneficiaries. In alignment with these findings, the Center for Medicaid and the Children’s Health Insurance Program (CHIP) Services (CMCS) recently issued subregulatory guidance encouraging states to cover and reimburse virtual interprofessional services [14]. This is a critical and necessary step to ensure specialist access for patients from all geographic and socioeconomic backgrounds. The endorsement for virtual interprofessional services reimbursement by this study and by CMCS, combined with the dearth of e-consultation platforms, presents a potential market opportunity for providers targeting Medicaid beneficiaries.

### Comparison to Prior Work

Recent reports by the GAO on Medicare and Medicaid detailed that the telehealth flexibility during the PHE period was enabled with the goal of lessening barriers affecting beneficiary care, augmenting provider availability, and ensuring adequate reimbursement to cover the additional costs of virtual care. Prior to the PHE, telehealth use had a strictly defined scope for Medicare and Medicaid reimbursement with gradual movement toward additional use based on emergent use cases.

The precedent for telehealth reimbursement is now being revisited due to the growing number of use cases and its potential to transform health care access, given its vital role during the COVID-19 pandemic. However, the barriers to universal adoption involve a perceived lack of research proving that telehealth is safe and effective on a broader scale.

Further reports by the MMDN have emphasized the importance of evidence as a key criterion for deciding reimbursement. They also noted that the impact of COVID-19 pandemic-related telehealth waivers on the quality of care remains unknown, with current broad evidence being inconclusive.

In the setting of these reports and prior precedent, our survey for MMDs explored clinical factors for continuing or discontinuing telehealth reimbursement which provides additional evidence to the discussion. The results showed that the primary factors influencing the responses were whether the service code could enhance access, outcomes, and equity of care. Conversely, the least common considerations were the level of evidence and financial maintainability of the service code. Our data highlight a discrepancy between prioritizing services that enhance access and outcomes, and those backed by a solid level of evidence.

Continuing the existing telehealth flexibility offers an opportunity to maintain equitable access and simultaneously expand the evidence base through additional measurement and analyses. With this study, we have identified, on a code-by-code basis, the services that MMDs find clinically appropriate for reimbursement while further evidence is gathered and evaluated.

### Cost and Technology Implementation

While some MMDs have expressed concerns about cost and implementation challenges associated with telehealth, safety-net providers are generally accepting of permanent telehealth adoption as long as there is sufficient reimbursement [9]. The evidence that Medicaid beneficiaries are eagerly willing to use telehealth services paired with appropriate Medicaid reimbursement can make the venture cost-effective for health centers in the long run. Studies evaluating telehealth cost-effectiveness have already proven to be efficient in certain areas, such as the management of type 2 diabetes and chronic heart failure when compared to usual methods of in-person care [15].

One approach to ease the implementation and cost burden for administrators and health centers is to default to full reimbursement for services in Figures 1 and 2 that have strong clinical support from MMDs. For services that did not have MMD support for reimbursement, state Medicaid programs can develop low-burden use management approaches with rapid cycle health services research to balance maintaining provisional and targeted coverage while being good stewards of state budgets.

### Policy Recommendations

A key workflow barrier that the COVID-19 PHE flexibility resolved was the originating site, or the physical location of the patient when using telehealth devices. Medicare and many Medicaid programs expanded the types of originating sites a patient could be at to include the home and other locations [9]. Waiver of the requirement for the home to be the originating site per section 1834(m) of the SSA is a requisite and foundational policy to enable the benefits of telehealth to be realized from an access, outcomes, and equity perspective. This PHE flexibility is due to expire in December 2024, which creates a legislative cliff that will imperil the most vulnerable Americans and wipe out the access gains achieved during the pandemic. To maintain the equitable access and outcomes gained during the pandemic the US Congress must create a legislative fix to permanently strike the SSA section 1834(m) restriction on the home as the originating site. If Congress cannot create a legislative fix before the end of 2024, then CMS needs to extend this flexibility to at least the end of 2025 to allow Congress to address the telehealth cliff. Additionally, CMCS could offer states waivers to the 1834(m) restriction on the home as the originating site through 1115 demonstrations or 1332 waivers.

In addition to addressing the 1834(m) restriction, CMCS should actualize the learnings from this study by issuing subregulatory guidance through a CMCS informational bulletin or state health official letter advising State Medicaid Directors to undertake the following steps: (1) fully reimburse telehealth services that are video and audio; (2) fully reimburse audio-only services that do not entirely require hands-on evaluation or intervention with the patient, such as telebehavioral health; (3) develop use management processes with a low-provider burden to consider full reimbursement of audio-only services that predominantly require hands-on evaluation or intervention with the patient while balancing access with safety and cost-effectiveness; and (4) promote the adoption of the newly created CPT 93 modifier for audio-only telehealth to ensure more research can be done on audio-only telehealth [16].

### CMCS Process Recommendations

To support the successful implementation of these policies, the CMCS approval processes for state plan amendments, waivers, and demonstrations should err on the side of enabling provider discretion and should be viewed through the lens of access, outcomes, and equity. The approval processes should also deeply involve the CMCS chief medical officer as well as the state-specific MMDs. Additionally, there should be standard quality measures that can be used across virtual and nonvirtual interventions.

### Limitations

First, our study had a primary limitation related to the size of our participant sample. Although our surveys were sent to all MMDs in the United States and our response rate was a reasonable 20% (n=10), the total sampling frame was small to begin with. We appreciate the support from AcademyHealth for distributing surveys and attempting follow-up with all the MMDs to optimize the response rate.

Second, within the survey itself, we acknowledge room for improvement. Specifically, question 2 could have been more explicit, addressing remote patient monitoring more directly. Additionally, we did not explicitly inquire about the preferred reimbursement levels for each service, so we can only draw limited conclusions about the precision of reimbursement.

Third, we encountered a technical issue during the initial survey distribution that hindered 2 respondents from selecting multiple options. Fortunately, we rectified this issue promptly but this technical issue may have led to incomplete responses by 2 respondents leading to their responses not being included in the analysis.

Finally, in terms of illness considerations within the survey, we intentionally excluded the assessment of illness severity as it was deemed beyond the scope of our study with respect to telehealth management.

### Conclusions

While telehealth existed before the COVID-19 outbreak, flexibility in telehealth policy has allowed for a more complete array of capabilities to be used across the United States. When considering the continuation of these policies, we validated our hypothesis that MMDs would be supportive of continued reimbursement of audio or video telehealth but would be wary of audio-only telehealth. The data show that hesitation for supporting audio-only telehealth was driven by clinical situations where visualization or hands-on intervention with the patient was necessary for diagnosis or treatment. There is also support for reimbursing remote monitoring services and telehealth interprofessional services, albeit with guardrails. These results are primarily from a perspective of improving access, outcomes, and equity; other state-specific factors such as fiscal impact and technical implementation may need to be taken into account when considering reimbursement decisions on telehealth.

These results on the clinical appropriateness of maintaining telehealth coverage should be taken in the context of broader research from the National Association of Medicaid Directors (NAMD) as well as the MMDN. The NAMD is conducting a parallel exercise with state Medicaid directors which offers a complementary perspective on the fiscal appropriateness and policy alignment of telehealth reimbursement. One way to strike a balance between equitably improving access and outcomes with fiscal stewardship is through accelerating value-based payment models in Medicaid. While state-specific decisions are being made on telehealth reimbursement, Congress must act to stave off the telehealth cliff.

## Appendix

Multimedia Appendix 1 Survey.

Multimedia Appendix 2 Current Procedural Terminology coding overview.

## Abbreviations

CHIP: Children’s Health Insurance Program
CMCS: Center for Medicaid and CHIP Services
CMS: Centers for Medicare & Medicaid Services
CPT: Current Procedural Terminology
GAO: Government Accountability Office
HCPCS: Healthcare Common Procedure Coding System
IRB: institutional review board
MMD: Medicaid medical director
MMDN: Medical Directors Network
NAMD: National Association of Medicaid Directors
PHE: public health emergency
SSA: Social Security Act

## Data Availability

The data sets generated and analyzed during this study are available from the corresponding author on reasonable request.
