# Supplementary figures and images for: Evaluation of Telehealth Services that are Clinically Appropriate for Reimbursement in the US Medicaid Population: Mixed Methods Study
Source: J Med Internet Res. 2024 Mar 28;26:e46412. doi: 10.2196/46412 (PMC11009844; doi:10.2196/46412)

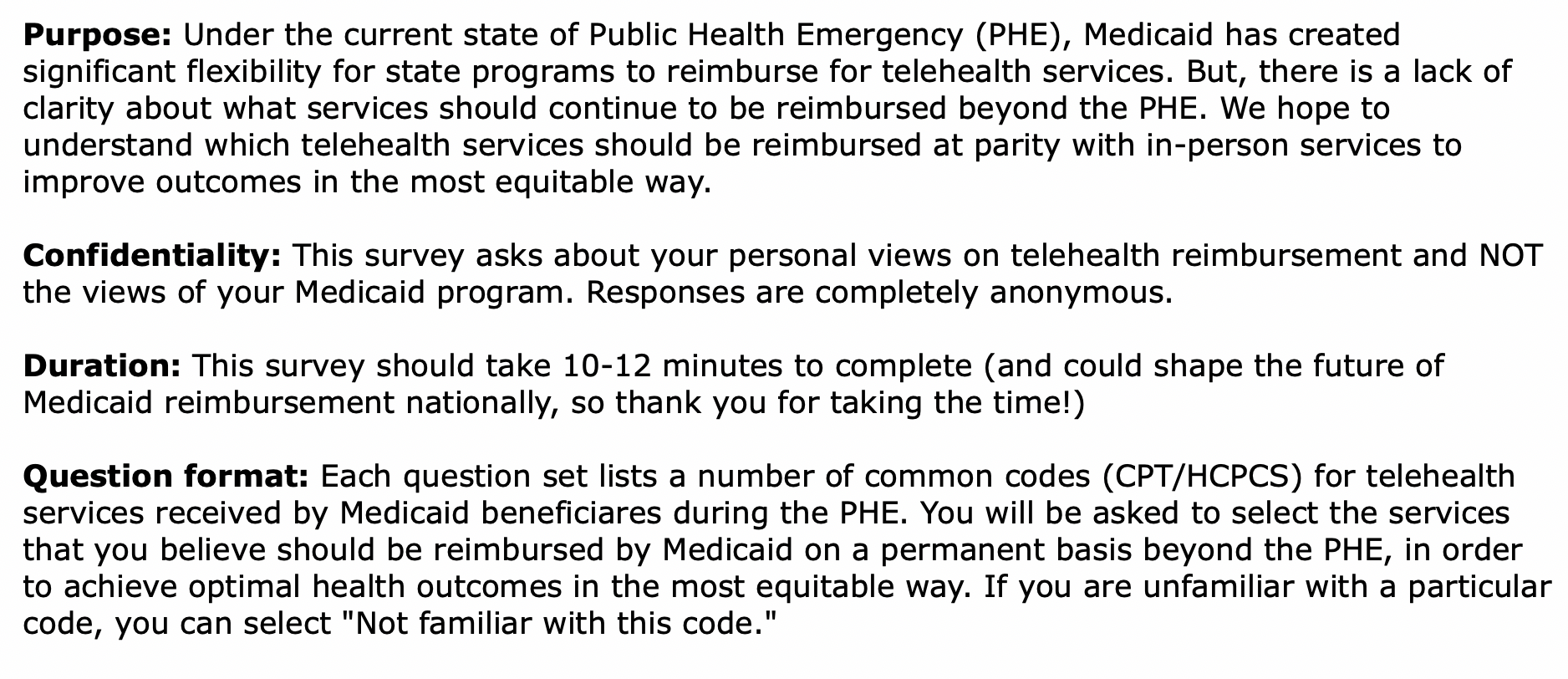


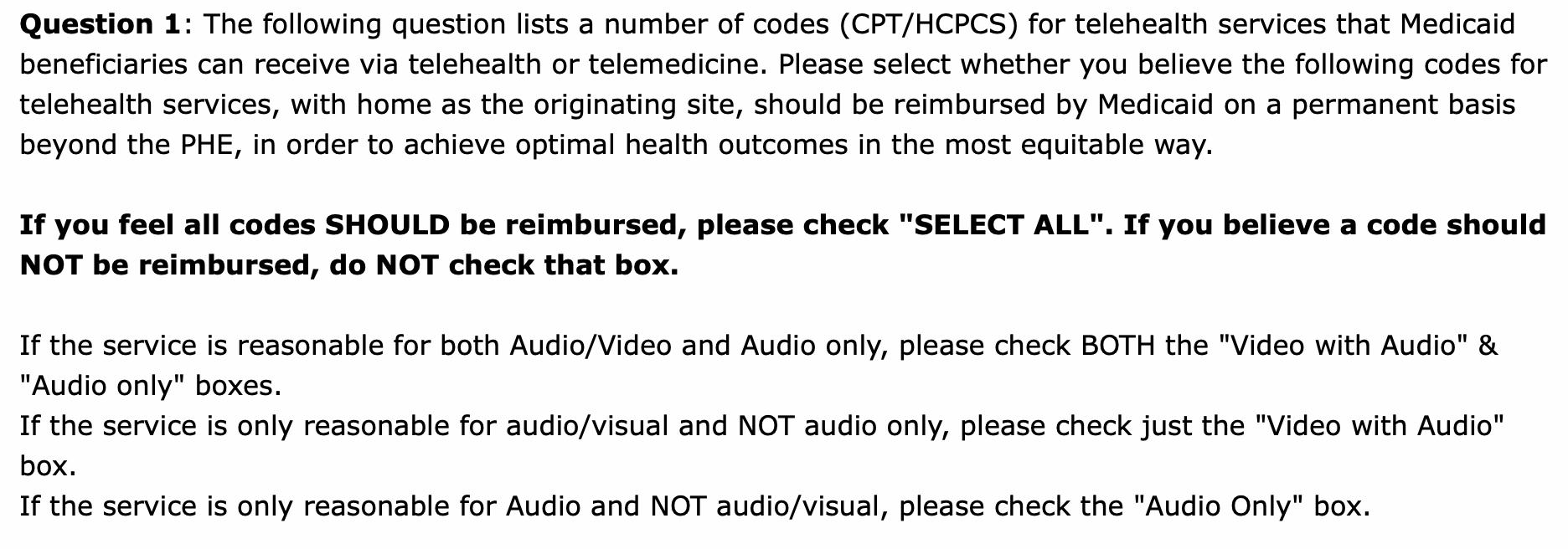


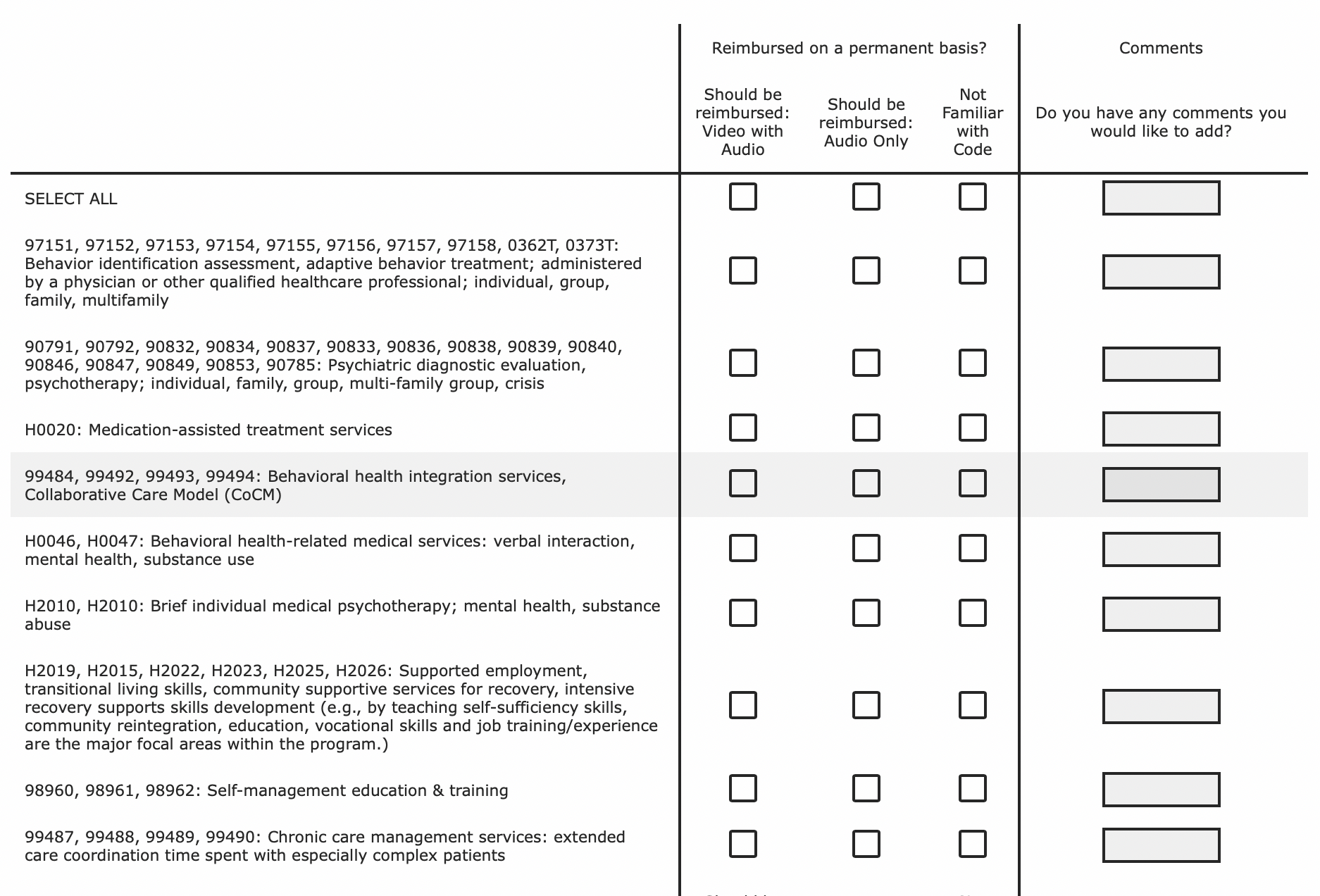


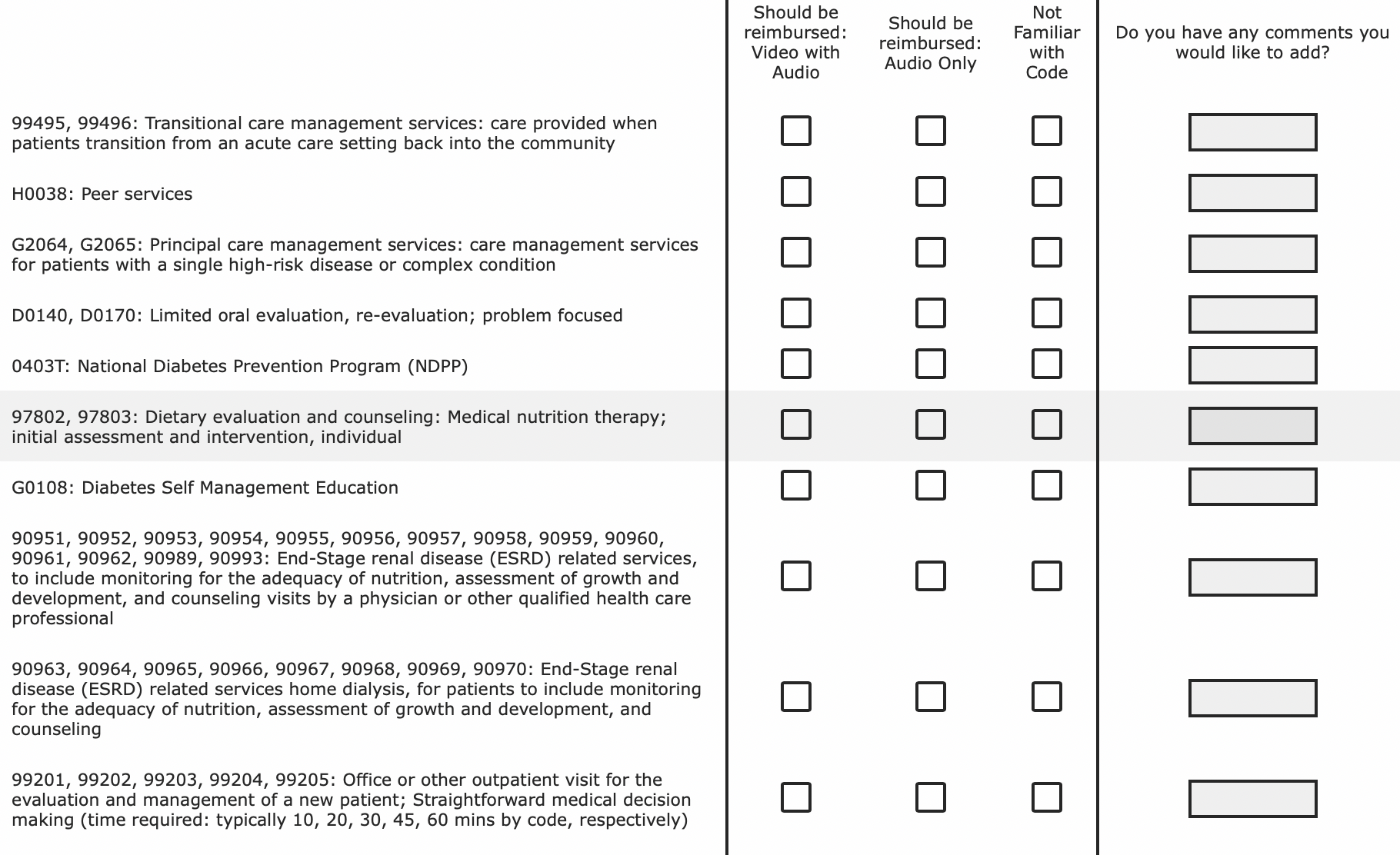


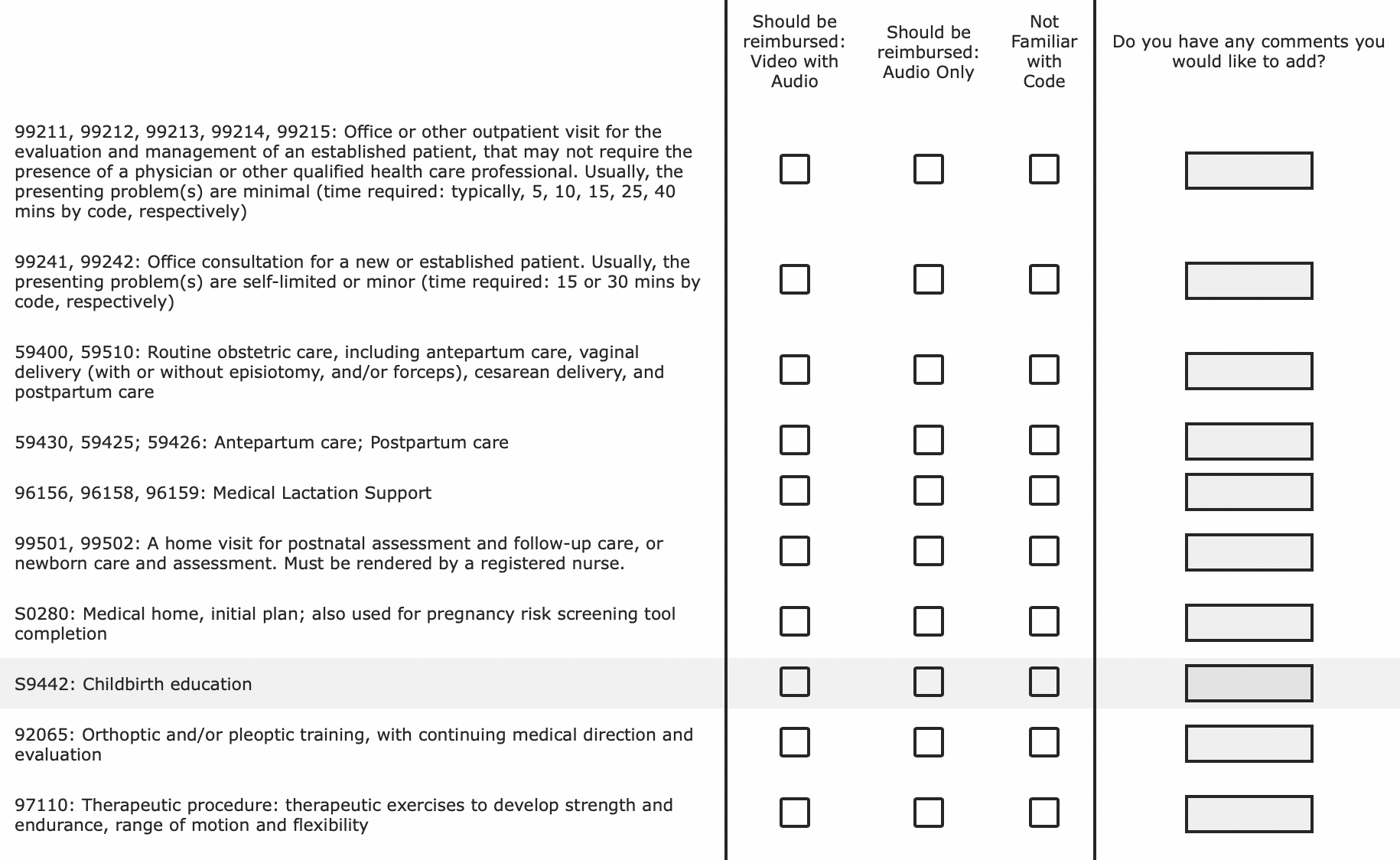


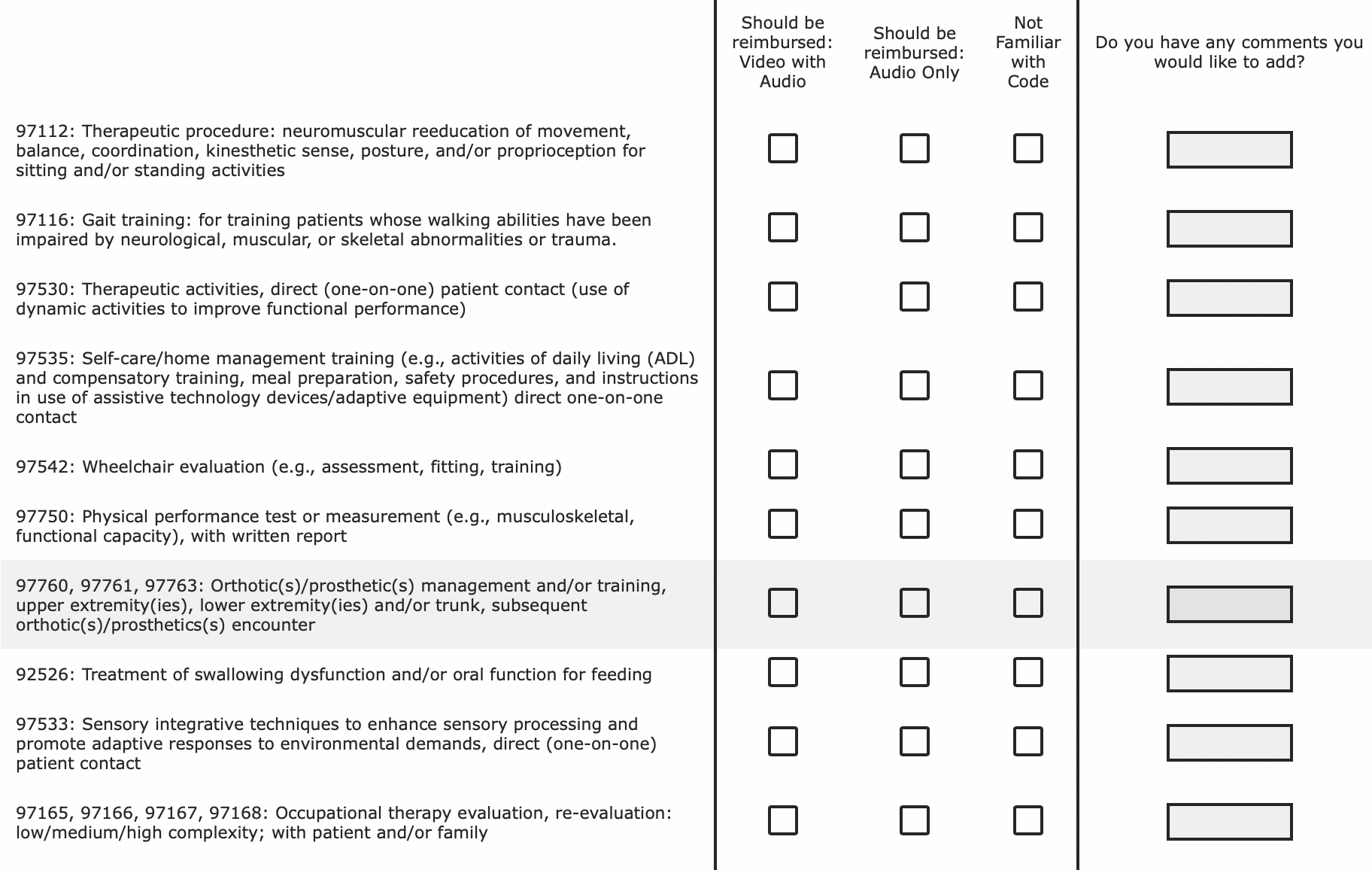


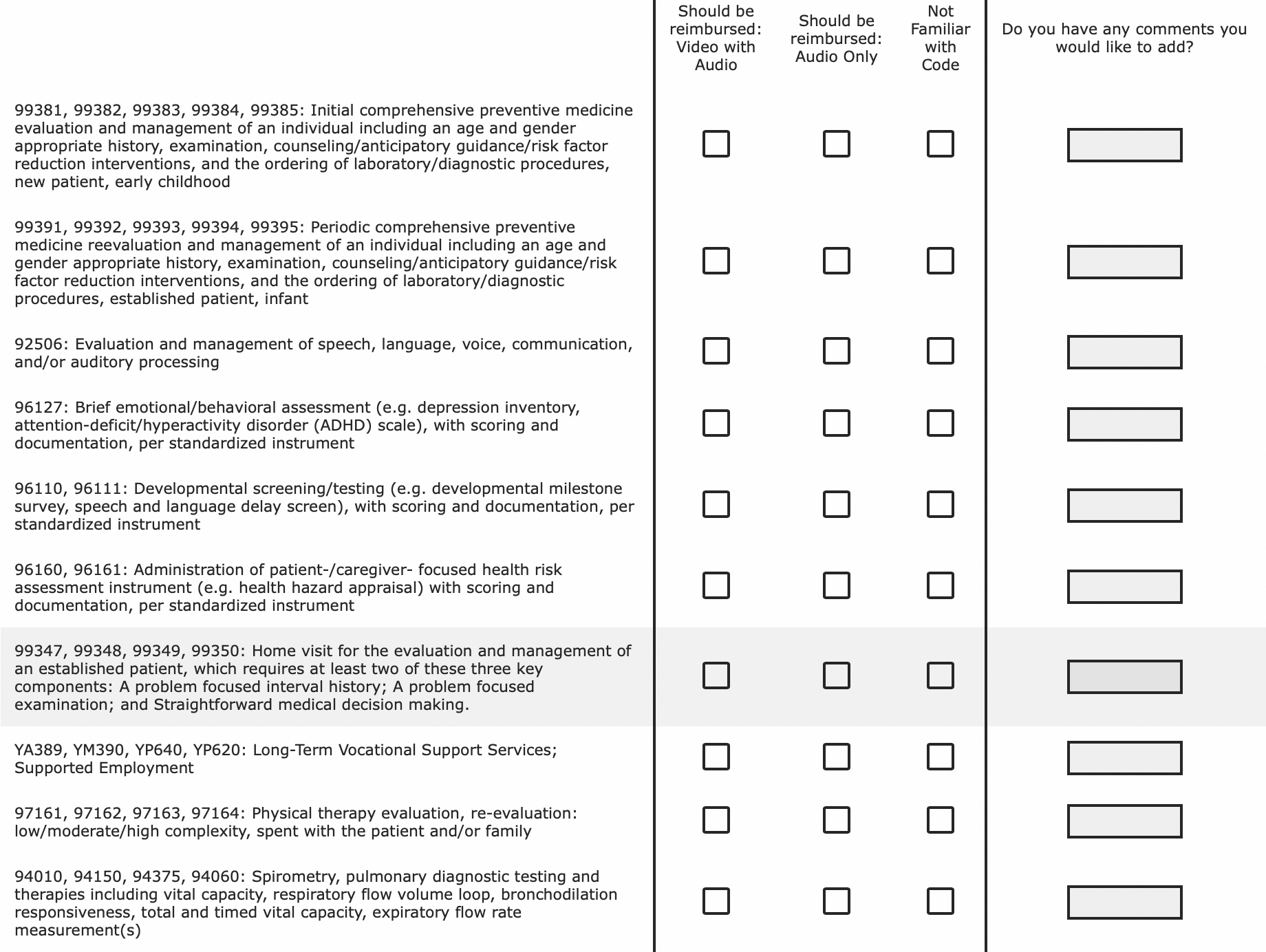


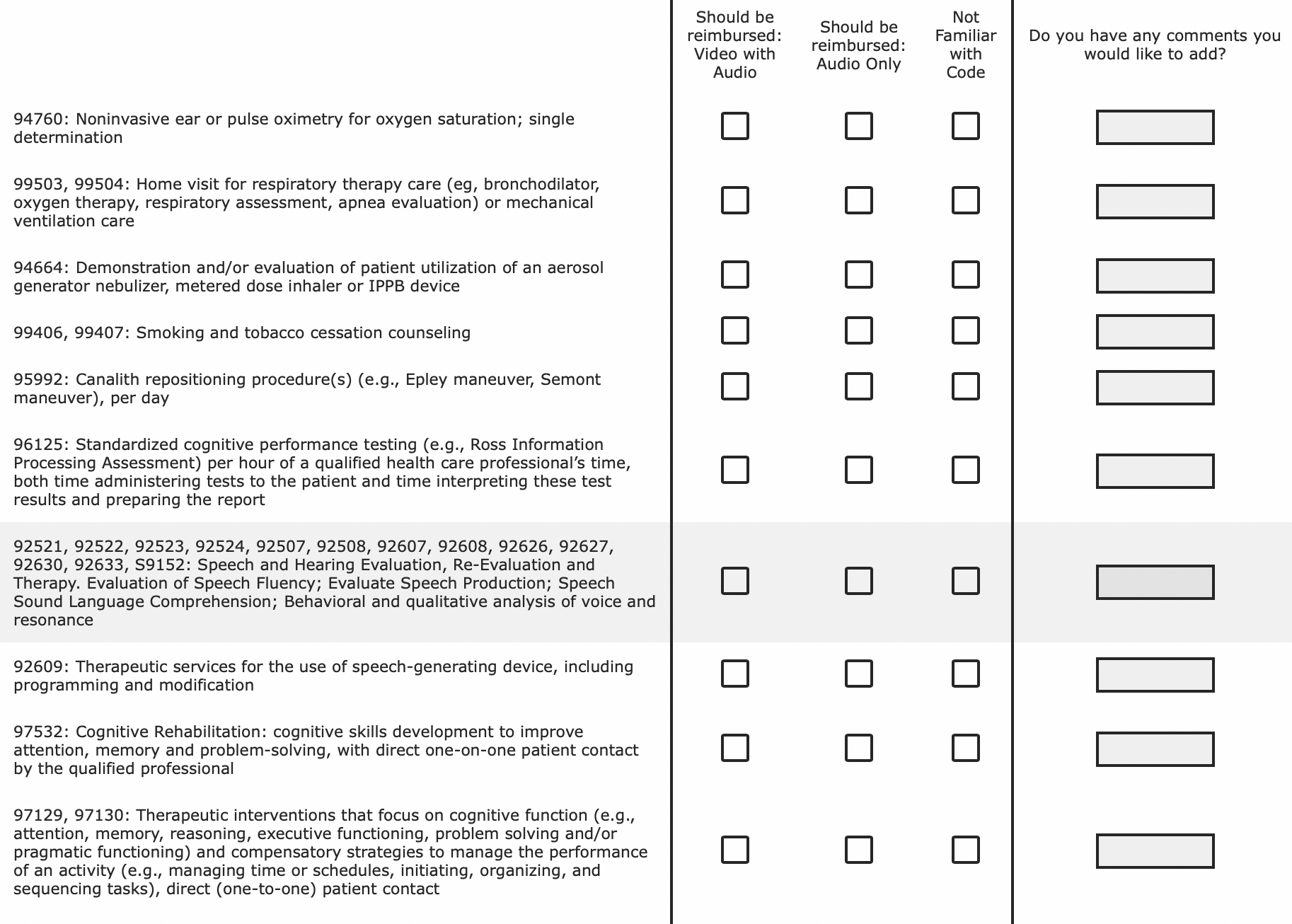


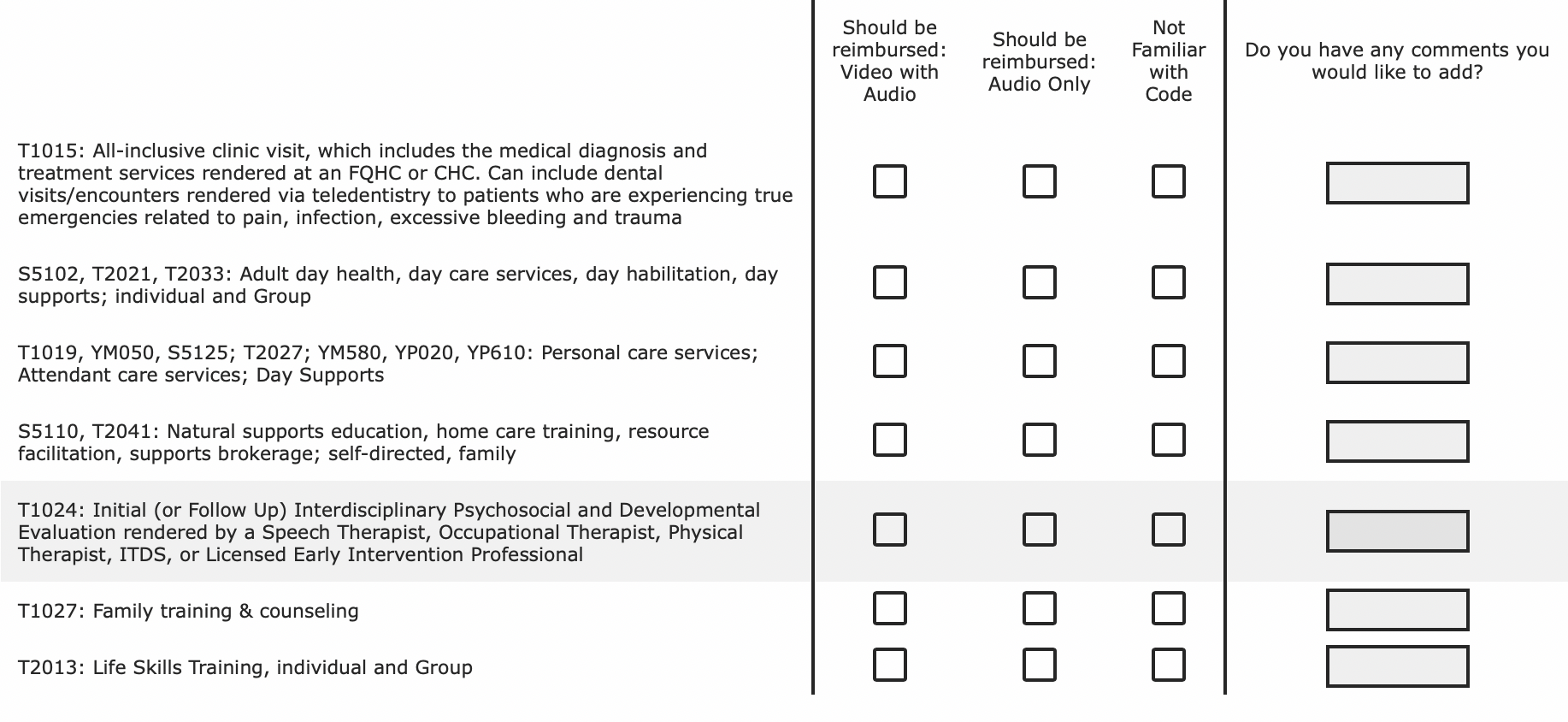


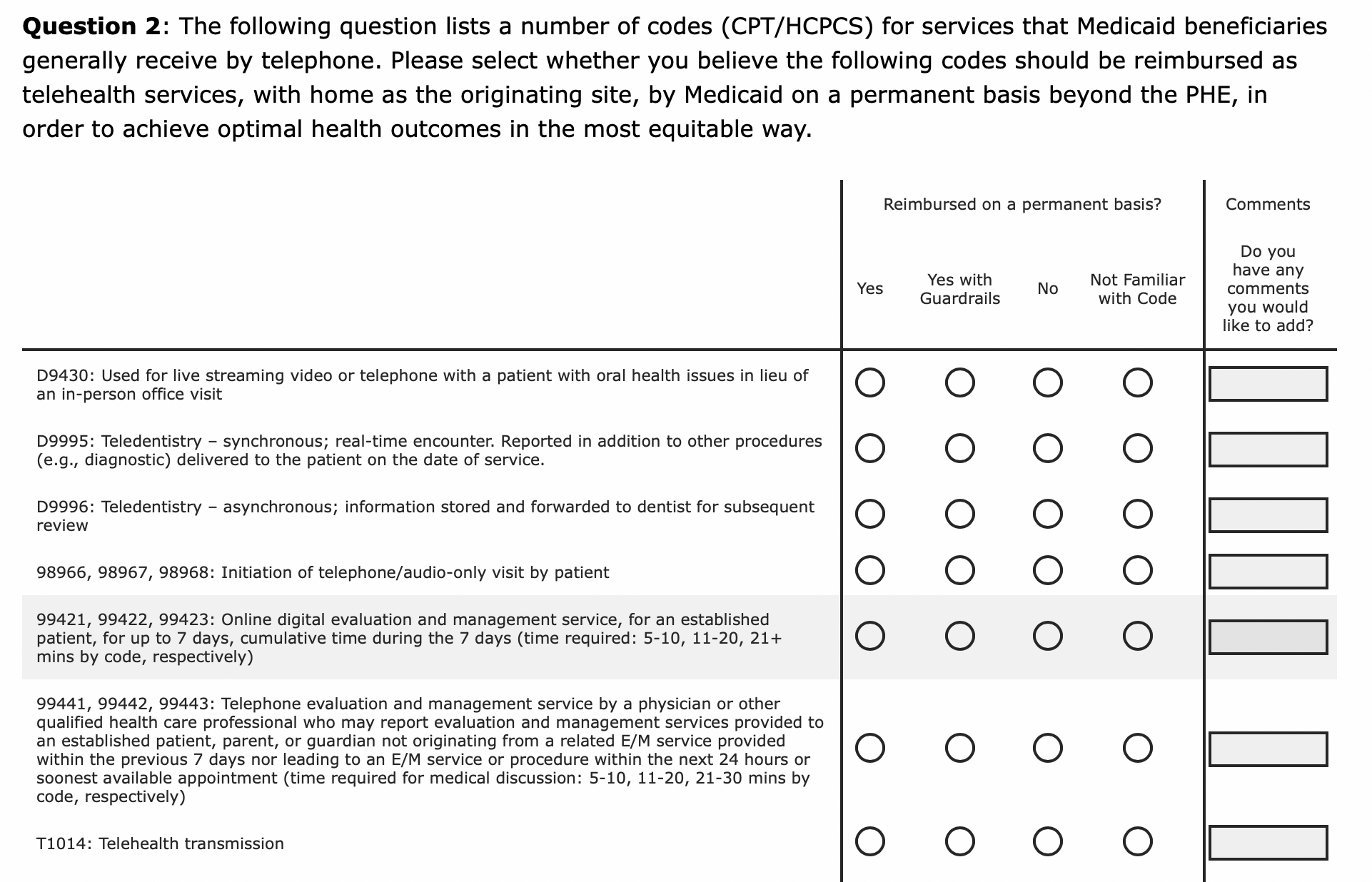


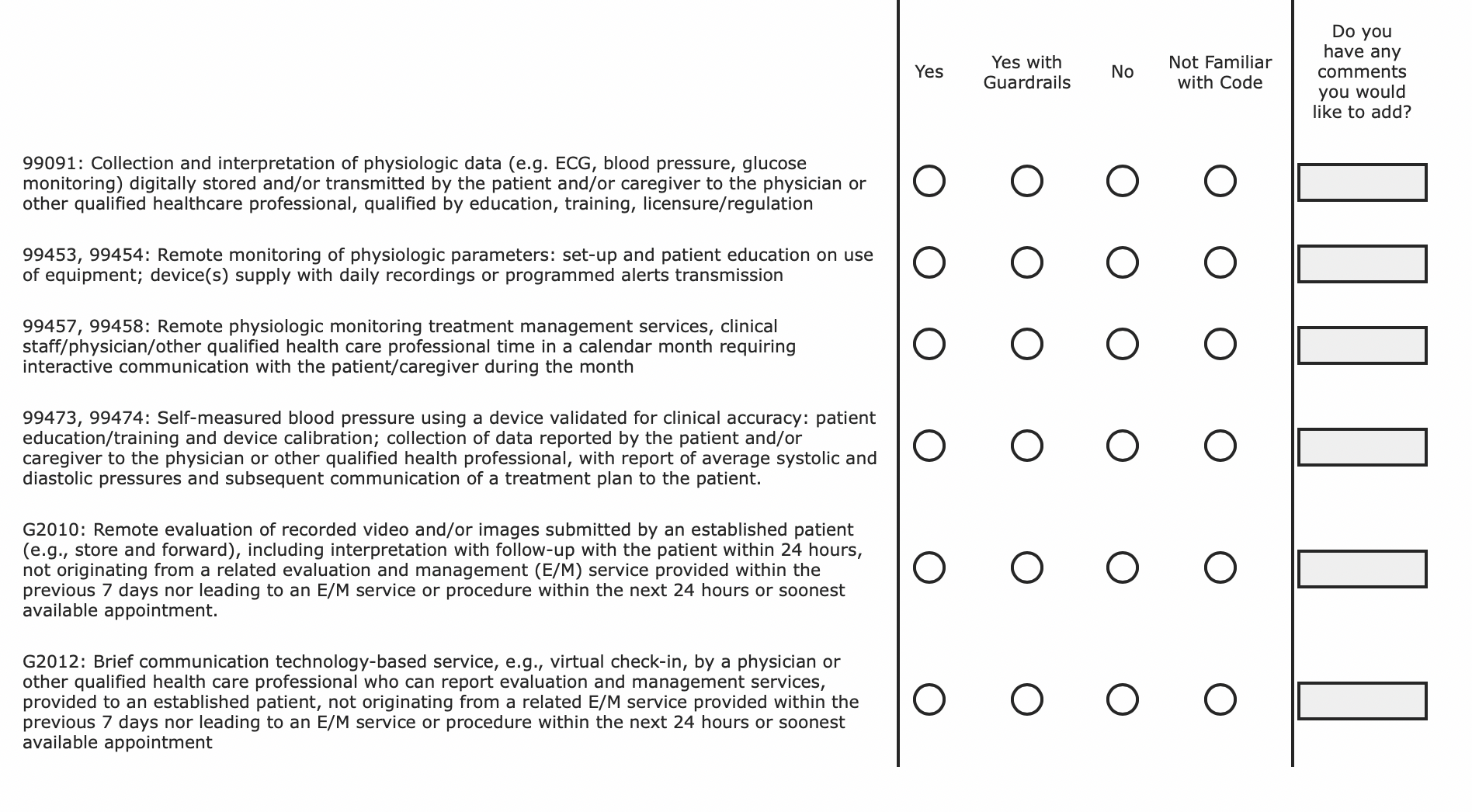


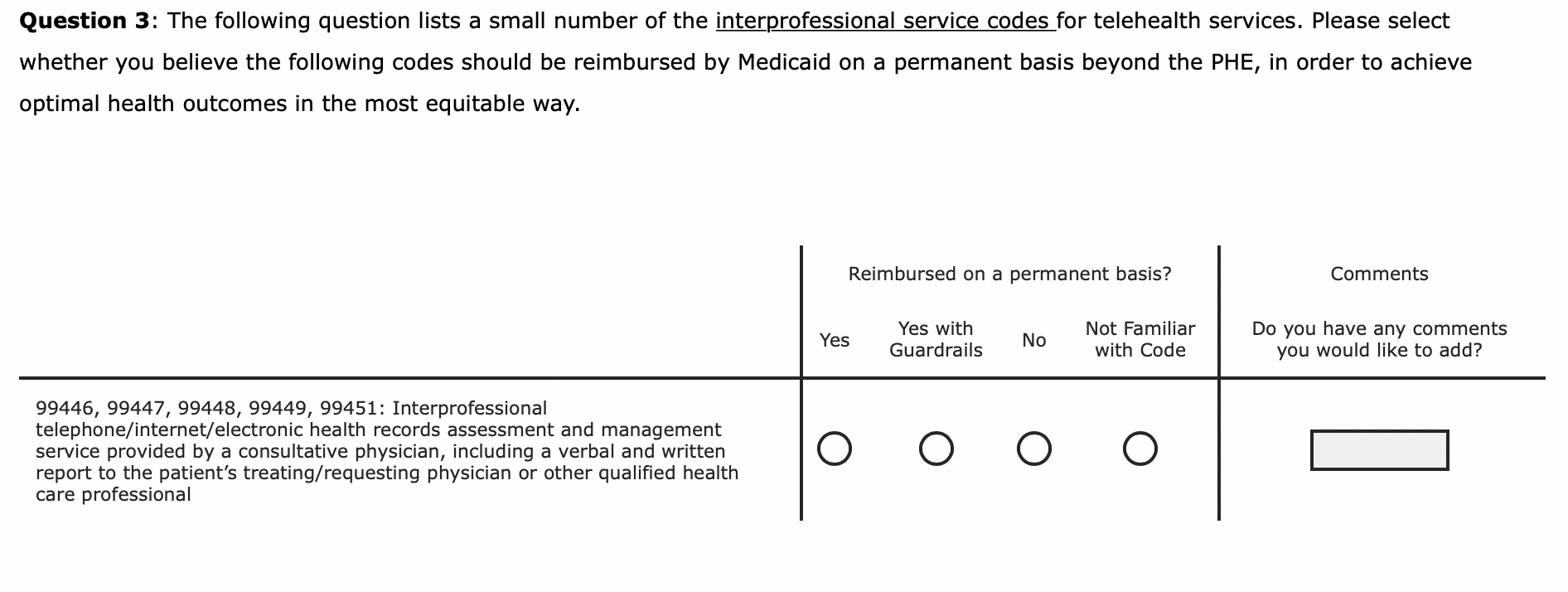


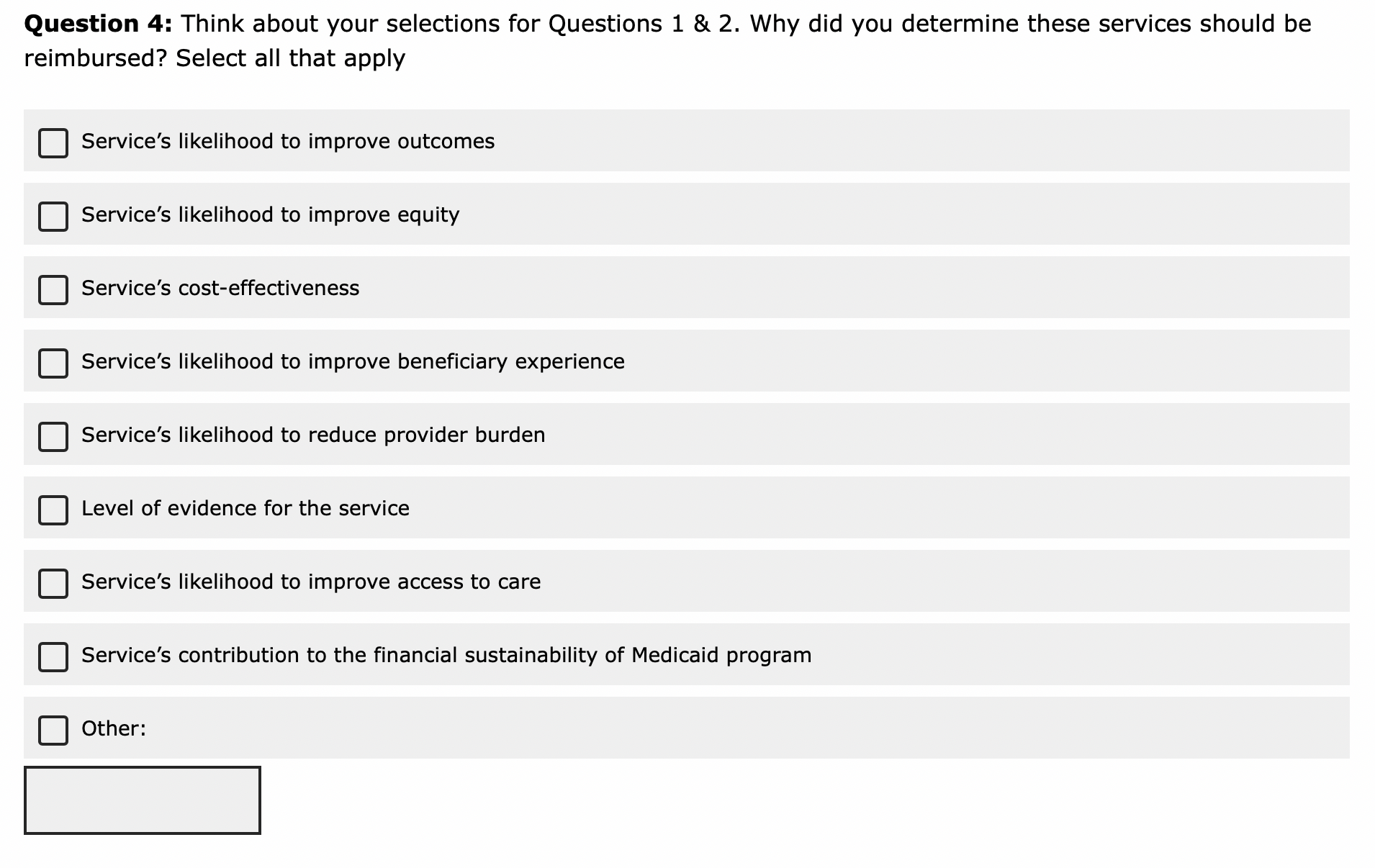

Supplement: Multimedia Appendix 1 [file jmir_v26i1e46412_app1.docx]
