# Supplementary material for: Evaluation of Telehealth Services that are Clinically Appropriate for Reimbursement in the US Medicaid Population: Mixed Methods Study
Source: J Med Internet Res. 2024 Mar 28;26:e46412. doi: 10.2196/46412 (PMC11009844; doi:10.2196/46412)
Supplement: Multimedia Appendix 2 [file jmir_v26i1e46412_app2.docx]

| **Category** | **CPT/HCPCS Code** | **CPT/HCPCS Description** | **Code & Description** |
| --- | --- | --- | --- |
| Audiology | 92630 | Auditory rehabilitation; prelingual hearing loss | 92630: Auditory rehabilitation; prelingual hearing loss |
| Audiology | 92633 | Auditory rehabilitation; postlingual hearing loss | 92633: Auditory rehabilitation; postlingual hearing loss |
| Behavioral Health | 90785 | Psychotherapy Complex Interactive Note: The use of this code telephonically is targeted to use of an interpreter, and not interactive play therapy. | 90785: Psychotherapy Complex Interactive Note: The use of this code telephonically is targeted to use of an interpreter, and not interactive play therapy. |
| Behavioral Health | 90791 | Psychiatric diagnostic evaluation | 90791: Psychiatric diagnostic evaluation |
| Behavioral Health | 90792 | Psychiatric diagnostic evaluation with medical services | 90792: Psychiatric diagnostic evaluation with medical services |
| Behavioral Health | 90839 | Psychotherapy Crisis Initial 60 minutes | 90839: Psychotherapy Crisis Initial 60 minutes |
| Behavioral Health | 90840 | Psychotherapy Crisis Each Additional 30 minutes | 90840: Psychotherapy Crisis Each Additional 30 minutes |
| Behavioral Health | 90849 | Multiple-Family Group Psychotherapy | 90849: Multiple-Family Group Psychotherapy |
| Behavioral Health | 90853 | Group Psychotherapy | 90853: Group Psychotherapy |
| Behavioral Health | H0020 | Medication-assisted treatment services | H0020: Medication-assisted treatment services |
| Behavioral Health | S5102 | Adult Day Health (TBI): Day care services, adult; per diem | S5102: Adult Day Health (TBI): Day care services, adult; per diem |
| Behavioral Health | S5125 | Personal Care (TBI): Attendant care services; per 15 minutes | S5125: Personal Care (TBI): Attendant care services; per 15 minutes |
| Behavioral Health | T1015 | all-inclusive clinic visit, which includes the medical diagnosis and treatment services rendered at an FQHC or CHC. Can include dental visits/encounters rendered via teledentistry to patients who are experiencing true emergencies related to pain, infection, excessive bleeding and trauma | T1015: all-inclusive clinic visit, which includes the medical diagnosis and treatment services rendered at an FQHC or CHC. Can include dental visits/encounters rendered via teledentistry to patients who are experiencing true emergencies related to pain, infection, excessive bleeding and trauma |
| Dentistry | D0170 | Re-evaluation – limited, problem focused. (established patient; not post-operative visit) Assessing the status of a previously existing condition. | D0170: Re-evaluation – limited, problem focused. (established patient; not post-operative visit) Assessing the status of a previously existing condition. |
| ESRD | 90951 | End-Stage renal disease (ESRD) related services monthly, for patients younger than 2 years of age to include monitoring for the adequacy of nutrition, assessment of growth and development, and counseling of parents with 4 or more face-to-face visits by a physician or other qualified health care professional per month. | 90951: End-Stage renal disease (ESRD) related services monthly, for patients younger than 2 years of age to include monitoring for the adequacy of nutrition, assessment of growth and development, and counseling of parents with 4 or more face-to-face visits by a physician or other qualified health care professional per month. |
| ESRD | 90952 | End-Stage renal disease (ESRD) related services monthly, for patients younger than 2 years of age to include monitoring for the adequacy of nutrition, assessment of growth and development, and counseling of parents with 2-3 face-to-face visits by a physician or other qualified health care professional per month. | 90952: End-Stage renal disease (ESRD) related services monthly, for patients younger than 2 years of age to include monitoring for the adequacy of nutrition, assessment of growth and development, and counseling of parents with 2-3 face-to-face visits by a physician or other qualified health care professional per month. |
| ESRD | 90953 | End-Stage renal disease (ESRD) related services monthly, for patients younger than 2 years of age to include monitoring for the adequacy of nutrition, assessment of growth and development, and counseling of parents with 1 face-to-face visit by a physician or other qualified health care professional per month. | 90953: End-Stage renal disease (ESRD) related services monthly, for patients younger than 2 years of age to include monitoring for the adequacy of nutrition, assessment of growth and development, and counseling of parents with 1 face-to-face visit by a physician or other qualified health care professional per month. |
| ESRD | 90954 | End-Stage renal disease (ESRD) related services monthly, for patients 2-11 years of age to include monitoring for the adequacy of nutrition, assessment of growth and development, and counseling of parents, with 4 or more face-to-face visits by a physician or other qualified health care professional per month. | 90954: End-Stage renal disease (ESRD) related services monthly, for patients 2-11 years of age to include monitoring for the adequacy of nutrition, assessment of growth and development, and counseling of parents, with 4 or more face-to-face visits by a physician or other qualified health care professional per month. |
| ESRD | 90955 | End-Stage renal disease (ESRD) related services monthly, for patients 2-11 years of age to include monitoring for the adequacy of nutrition, assessment of growth and development, and counseling of parents with 2-3 face-to-face visits by a physician or other qualified health care professional per month. | 90955: End-Stage renal disease (ESRD) related services monthly, for patients 2-11 years of age to include monitoring for the adequacy of nutrition, assessment of growth and development, and counseling of parents with 2-3 face-to-face visits by a physician or other qualified health care professional per month. |
| ESRD | 90956 | End-Stage renal disease (ESRD) related services monthly, for patients 2-11 years of age to include monitoring for the adequacy of nutrition, assessment of growth and development, and counseling of parents, with 1 face-to-face visit by a physician or other qualified health care professional per month. | 90956: End-Stage renal disease (ESRD) related services monthly, for patients 2-11 years of age to include monitoring for the adequacy of nutrition, assessment of growth and development, and counseling of parents, with 1 face-to-face visit by a physician or other qualified health care professional per month. |
| ESRD | 90957 | End-Stage renal disease (ESRD) related services monthly, for patients 12-19 years of age to include monitoring for the adequacy of nutrition, assessment of growth and development, and counseling of parents with 4 or more face-to-face visits by a physician or other qualified health care professional per month. | 90957: End-Stage renal disease (ESRD) related services monthly, for patients 12-19 years of age to include monitoring for the adequacy of nutrition, assessment of growth and development, and counseling of parents with 4 or more face-to-face visits by a physician or other qualified health care professional per month. |
| ESRD | 90958 | End-Stage renal disease (ESRD) related services monthly, for patients 12-19 years of age to include monitoring for the adequacy of nutrition, assessment of growth and development, and counseling of parents with 2-3 face-to-face visits by a physician or other qualified health care professional per month. | 90958: End-Stage renal disease (ESRD) related services monthly, for patients 12-19 years of age to include monitoring for the adequacy of nutrition, assessment of growth and development, and counseling of parents with 2-3 face-to-face visits by a physician or other qualified health care professional per month. |
| ESRD | 90959 | End-Stage renal disease (ESRD) related services monthly, for patients 12-19 years of age to include monitoring for the adequacy of nutrition, assessment of growth and development, and counseling of parents with 1 face-to-face visit by a physician or other qualified health care professional per month. | 90959: End-Stage renal disease (ESRD) related services monthly, for patients 12-19 years of age to include monitoring for the adequacy of nutrition, assessment of growth and development, and counseling of parents with 1 face-to-face visit by a physician or other qualified health care professional per month. |
| ESRD | 90960 | End-Stage renal disease (ESRD) related services monthly, for patients 20 years of age and older; with 4 or more face-to-face visits by a physician or other qualified health care professional per month. | 90960: End-Stage renal disease (ESRD) related services monthly, for patients 20 years of age and older; with 4 or more face-to-face visits by a physician or other qualified health care professional per month. |
| ESRD | 90961 | End-Stage renal disease (ESRD) related services monthly, for patients 20 years of age and older; with 2-3 face-to-face visits by a physician or other qualified health care professional per month. | 90961: End-Stage renal disease (ESRD) related services monthly, for patients 20 years of age and older; with 2-3 face-to-face visits by a physician or other qualified health care professional per month. |
| ESRD | 90962 | End-Stage renal disease (ESRD) related services monthly, for patients 20 years of age and older; with 1 face-to-face visit by a physician or other qualified health care professional per month. | 90962: End-Stage renal disease (ESRD) related services monthly, for patients 20 years of age and older; with 1 face-to-face visit by a physician or other qualified health care professional per month. |
| ESRD | 90963 | End-Stage renal disease (ESRD) related services for home dialysis per full month, for patients younger than 2 years of age to include monitoring for the adequacy of nutrition, assessment of growth and development, and counseling of parents. | 90963: End-Stage renal disease (ESRD) related services for home dialysis per full month, for patients younger than 2 years of age to include monitoring for the adequacy of nutrition, assessment of growth and development, and counseling of parents. |
| ESRD | 90964 | End-Stage renal disease (ESRD) related services for home dialysis per full month, for patients 2-11 years of age to include monitoring for the adequacy of nutrition, assessment of growth and development, and counseling of parents. | 90964: End-Stage renal disease (ESRD) related services for home dialysis per full month, for patients 2-11 years of age to include monitoring for the adequacy of nutrition, assessment of growth and development, and counseling of parents. |
| ESRD | 90965 | End-Stage renal disease (ESRD) related services for home dialysis per full month, for patients 12-19 years of age to include monitoring for the adequacy of nutrition, assessment of growth and development, and counseling of parents. | 90965: End-Stage renal disease (ESRD) related services for home dialysis per full month, for patients 12-19 years of age to include monitoring for the adequacy of nutrition, assessment of growth and development, and counseling of parents. |
| ESRD | 90966 | End-Stage renal disease (ESRD) related services for home dialysis per full month, for patients 20 years of age and older. | 90966: End-Stage renal disease (ESRD) related services for home dialysis per full month, for patients 20 years of age and older. |
| ESRD | 90967 | End-Stage renal disease (ESRD) related services for dialysis less than a full month of service, per day, for patients younger than 2 years old. | 90967: End-Stage renal disease (ESRD) related services for dialysis less than a full month of service, per day, for patients younger than 2 years old. |
| ESRD | 90968 | End-Stage renal disease (ESRD) related services for dialysis less than a full month of service, per day, for patients 2-11 years of age. | 90968: End-Stage renal disease (ESRD) related services for dialysis less than a full month of service, per day, for patients 2-11 years of age. |
| ESRD | 90969 | End-Stage renal disease (ESRD) related services for dialysis less than a full month of service, per day, for patients 12-19 years of age. | 90969: End-Stage renal disease (ESRD) related services for dialysis less than a full month of service, per day, for patients 12-19 years of age. |
| ESRD | 90970 | End-Stage renal disease (ESRD) related services for dialysis less than a full month of service, per day, for patients 20 years of age and older. | 90970: End-Stage renal disease (ESRD) related services for dialysis less than a full month of service, per day, for patients 20 years of age and older. |
| ESRD | 90989 | Dialysis training, patient, including helper where applicable, any mode, completed course. | 90989: Dialysis training, patient, including helper where applicable, any mode, completed course. |
| ESRD | 90993 | Dialysis training, patient, including helper where applicable, any mode, course not completed, per training session. | 90993: Dialysis training, patient, including helper where applicable, any mode, course not completed, per training session. |
| Maternal and Perinatal Health | 59400 | Routine obstetric care, including antepartum care, vaginal delivery (with or without episiotomy, and/or forceps) and postpartum care | 59400: Routine obstetric care, including antepartum care, vaginal delivery (with or without episiotomy, and/or forceps) and postpartum care |
| Maternal and Perinatal Health | 59425 | Antepartum care only; 4-6 visits | 59425: Antepartum care only; 4-6 visits |
| Maternal and Perinatal Health | 59426 | Antepartum care only; 7 or more visits | 59426: Antepartum care only; 7 or more visits |
| Maternal and Perinatal Health | 59430 | Postpartum care only; separate procedure | 59430: Postpartum care only; separate procedure |
| Maternal and Perinatal Health | 59510 | Routine obstetric care including antepartum care, cesarean delivery, and postpartum care | 59510: Routine obstetric care including antepartum care, cesarean delivery, and postpartum care |
| Occupational Therapy | 92065 | Orthoptic and/or pleoptic training, with continuing medical direction and evaluation | 92065: Orthoptic and/or pleoptic training, with continuing medical direction and evaluation |
| Occupational Therapy | 97110 | Therapeutic procedure, 1 or more areas, each 15 minutes; therapeutic exercises to develop strength and endurance, range of motion and flexibility | 97110: Therapeutic procedure, 1 or more areas, each 15 minutes; therapeutic exercises to develop strength and endurance, range of motion and flexibility |
| Occupational Therapy | 97112 | Therapeutic procedure, 1 or more areas, each 15 minutes; neuromuscular reeducation of movement, balance, coordination, kinesthetic sense, posture, and/or proprioception for sitting and/or standing activities | 97112: Therapeutic procedure, 1 or more areas, each 15 minutes; neuromuscular reeducation of movement, balance, coordination, kinesthetic sense, posture, and/or proprioception for sitting and/or standing activities |
| Occupational Therapy | 97116 | Gait training: for training patients whose walking abilities have been impaired by neurological, muscular, or skeletal abnormalities or trauma.Therapeutic procedure, 1 or more areas, each 15 minutes; gait training (includes stair climbing) | 97116: Gait training: for training patients whose walking abilities have been impaired by neurological, muscular, or skeletal abnormalities or trauma.Therapeutic procedure, 1 or more areas, each 15 minutes; gait training (includes stair climbing) |
| Occupational Therapy | 97530 | Therapeutic activities, direct (one-on-one) patient contact (use of dynamic activities to improve functional performance), each 15 minutes | 97530: Therapeutic activities, direct (one-on-one) patient contact (use of dynamic activities to improve functional performance), each 15 minutes |
| Occupational Therapy | 97535 | Self-care/home management training (e.g., activities of daily living (ADL) and compensatory training, meal preparation, safety procedures, and instructions in use of assistive technology devices/adaptive equipment) direct one-on-one contact, each 15 minutes | 97535: Self-care/home management training (e.g., activities of daily living (ADL) and compensatory training, meal preparation, safety procedures, and instructions in use of assistive technology devices/adaptive equipment) direct one-on-one contact, each 15 minutes |
| Occupational Therapy | 97542 | Wheelchair evaluation (e.g., assessment, fitting, training), each 15 minutes | 97542: Wheelchair evaluation (e.g., assessment, fitting, training), each 15 minutes |
| Occupational Therapy | 97750 | Physical performance test or measurement (e.g., musculoskeletal, functional capacity), with written report, each 15 minutes. | 97750: Physical performance test or measurement (e.g., musculoskeletal, functional capacity), with written report, each 15 minutes. |
| Occupational Therapy | 97760 97761 97763 | Orthotic(s)/prosthetic(s) management and/or training, upper extremity(ies), lower extremity(ies) and/or trunk, subsequent orthotic(s)/prosthetics(s) encounter, each 15 minutes | 97760 97761 97763: Orthotic(s)/prosthetic(s) management and/or training, upper extremity(ies), lower extremity(ies) and/or trunk, subsequent orthotic(s)/prosthetics(s) encounter, each 15 minutes |
| Outpatient Specialized Therapy | 95992 | Canalith repositioning procedure(s) (e.g., Epley maneuver, Semont maneuver), per day | 95992: Canalith repositioning procedure(s) (e.g., Epley maneuver, Semont maneuver), per day |
| Pediatrics | 0362T | Behavior identification supporting assessment, each 15 minutes of technicians’ time face-to-face with a patient, requiring the following components: • administered by the physician or other qualified healthcare professional who is on site4 ; • with the assistance of two or more technicians; • for a patient who exhibits destructive behavior; • completed in an environment that is customized to the patient’s behavior | 0362T: Behavior identification supporting assessment, each 15 minutes of technicians’ time face-to-face with a patient, requiring the following components: • administered by the physician or other qualified healthcare professional who is on site4 ; • with the assistance of two or more technicians; • for a patient who exhibits destructive behavior; • completed in an environment that is customized to the patient’s behavior |
| Pediatrics | 0373T | Adaptive behavior treatment with protocol modification, each 15 minutes of technicians’ time face-toface with a patient, requiring the following components: • administered by the physician or other qualified healthcare professional who is on site4 ; • with the assistance of two or more technicians; • for a patient who exhibits destructive behavior; • completed in an environment that is customized, to the patient’s behavior. | 0373T: Adaptive behavior treatment with protocol modification, each 15 minutes of technicians’ time face-toface with a patient, requiring the following components: • administered by the physician or other qualified healthcare professional who is on site4 ; • with the assistance of two or more technicians; • for a patient who exhibits destructive behavior; • completed in an environment that is customized, to the patient’s behavior. |
| Personal care and habilitation | T1019 U4 | Individual Support: Personal care services, per 15 minutes, not for an inpatient or resident of a hospital, nursing facility, icf/mr or imd, part of the individualized plan of treatment (code may not be used to identify services provided by home health aide or certified nurse assistant) | T1019 U4: Individual Support: Personal care services, per 15 minutes, not for an inpatient or resident of a hospital, nursing facility, icf/mr or imd, part of the individualized plan of treatment (code may not be used to identify services provided by home health aide or certified nurse assistant) |
| Personal care and habilitation | T2021 T2021 HQ | Day habilitation, Day Supports - Individual and Group (Innovations and TBI) | T2021 T2021 HQ: Day habilitation, Day Supports - Individual and Group (Innovations and TBI) |
| Personal care and habilitation | T2027 | Day Supports - Developmental Day (Specialized childcare, waiver; per 15 minutes) | T2027: Day Supports - Developmental Day (Specialized childcare, waiver; per 15 minutes) |
| Personal care and habilitation | YM050 | Personal Care Services | YM050: Personal Care Services |
| Personal care and habilitation | YM580 | Day Supports: allows for all supports services provided on behalf of an individual in a day setting to be delivered under one service heading and reported in an aggregate daily record. Individual services which may be included in this service are those generally understood as habilitation/ support services: Assistance with acquisition, retention, or improvement in self-help, socialization and adaptive skills which takes place in a non-residential setting, separate from the home or facility in which the individual resides. Services shall normally be furnished four (4) or more hours per day on a regularly scheduled basis, for one (1) or more days per week unless provided as an adjunct to other day activities included in an individual’s plan of care | YM580: Day Supports: allows for all supports services provided on behalf of an individual in a day setting to be delivered under one service heading and reported in an aggregate daily record. Individual services which may be included in this service are those generally understood as habilitation/ support services: Assistance with acquisition, retention, or improvement in self-help, socialization and adaptive skills which takes place in a non-residential setting, separate from the home or facility in which the individual resides. Services shall normally be furnished four (4) or more hours per day on a regularly scheduled basis, for one (1) or more days per week unless provided as an adjunct to other day activities included in an individual’s plan of care |
| Personal care and habilitation | YP020 - Ind | Personal Assistance: support service which provides aid to a client so that the client can engage in activities and interactions from which the client would otherwise be limited or excluded because of his disability or disabilities. The assistance includes: (1) assistance in personal or regular living activities in the client's home, (2) support in skill development, or (3) support and accompaniment of the client in regular community activities or in specialized treatment, habilitation or rehabilitation service programs. | YP020 - Ind: Personal Assistance: support service which provides aid to a client so that the client can engage in activities and interactions from which the client would otherwise be limited or excluded because of his disability or disabilities. The assistance includes: (1) assistance in personal or regular living activities in the client's home, (2) support in skill development, or (3) support and accompaniment of the client in regular community activities or in specialized treatment, habilitation or rehabilitation service programs. |
| Personal care and habilitation | YP610 | Developmental Day: Developmental Day is a day/night service which provides individual habilitative programming for children with intellectual/developmental disabilities, with or at risk for developmental disabilities, or atypical development in special licensed child care center. It is designed to meet the developmental needs of the children in an inclusive setting to promote skill acquisition in areas such as self-help, fine and gross motor skills, language and communication, cognitive and social skills in order to facilitate their functioning in a less restrictive environment. This service is also designed to meet child care needs of families and to provide family training and support. | YP610: Developmental Day: Developmental Day is a day/night service which provides individual habilitative programming for children with intellectual/developmental disabilities, with or at risk for developmental disabilities, or atypical development in special licensed child care center. It is designed to meet the developmental needs of the children in an inclusive setting to promote skill acquisition in areas such as self-help, fine and gross motor skills, language and communication, cognitive and social skills in order to facilitate their functioning in a less restrictive environment. This service is also designed to meet child care needs of families and to provide family training and support. |
| Personal care and habilitation | YP620 | Adult Developmental Vocational Program (ADVP): An Adult Developmental Vocational Program (ADVP) is a day/night service which provides organized developmental activities for individuals with intellectual/developmental disabilities to prepare the individual to live and work as independently as possible. The activities and services of ADVP are designed to adhere to the principles of normalization and community integration. | YP620: Adult Developmental Vocational Program (ADVP): An Adult Developmental Vocational Program (ADVP) is a day/night service which provides organized developmental activities for individuals with intellectual/developmental disabilities to prepare the individual to live and work as independently as possible. The activities and services of ADVP are designed to adhere to the principles of normalization and community integration. |
| Respiratory | 94010 | Spirometry, including graphic record, total and timed vital capacity, expiratory flow rate measurement(s), with or without maximal voluntary ventilation | 94010: Spirometry, including graphic record, total and timed vital capacity, expiratory flow rate measurement(s), with or without maximal voluntary ventilation |
| Respiratory | 94060 | Bronchodilation responsiveness, spirometry as in 94010, pre- and post-bronchodilator administration | 94060: Bronchodilation responsiveness, spirometry as in 94010, pre- and post-bronchodilator administration |
| Respiratory | 94150 | Vital capacity, total (separate procedure) | 94150: Vital capacity, total (separate procedure) |
| Respiratory | 94375 | Respiratory flow volume loop | 94375: Respiratory flow volume loop |
| Respiratory | 94760 | Noninvasive ear or pulse oximetry for oxygen saturation; single determination | 94760: Noninvasive ear or pulse oximetry for oxygen saturation; single determination |
| Respiratory | 99503 | Home visit for respiratory therapy care (eg, bronchodilator, oxygen therapy, respiratory assessment, apnea evaluation) | 99503: Home visit for respiratory therapy care (eg, bronchodilator, oxygen therapy, respiratory assessment, apnea evaluation) |
| Respiratory | 99504 | Home visit for mechanical ventilation care | 99504: Home visit for mechanical ventilation care |
| Speech Therapy | 92609 | Therapeutic services for the use of speech-generating device, including programming and modification | 92609: Therapeutic services for the use of speech-generating device, including programming and modification |
| Speech Therapy | 92626 | Evaluation of auditory rehabilitation status; first hour | 92626: Evaluation of auditory rehabilitation status; first hour |
| Speech Therapy | 92627 | Evaluation of auditory rehabilitation status; additional 15 min | 92627: Evaluation of auditory rehabilitation status; additional 15 min |
| Speech Therapy | 92630 | Aud Rehab Pre-Ling Hear Loss | 92630: Aud Rehab Pre-Ling Hear Loss |
| Speech Therapy | 92633 | Aud Rehab Postling Hear Loss | 92633: Aud Rehab Postling Hear Loss |
| Speech Therapy | 96125 | Standardized cognitive performance testing (e.g., Ross Information Processing Assessment) per hour of a qualified health care professional’s time, both face-to-face time administering tests to the patient and time interpreting these test results and preparing the report | 96125: Standardized cognitive performance testing (e.g., Ross Information Processing Assessment) per hour of a qualified health care professional’s time, both face-to-face time administering tests to the patient and time interpreting these test results and preparing the report |
| Behavioral Health | 90832 | Psychotherapy, 30 minutes with patient | 90832: Psychotherapy, 30 minutes with patient |
| Behavioral Health | 90833 | Psychotherapy, 30 minutes with patient when performed with an evaluation and management service | 90833: Psychotherapy, 30 minutes with patient when performed with an evaluation and management service |
| Behavioral Health | 90834 | Psychotherapy, 45 minutes with patient | 90834: Psychotherapy, 45 minutes with patient |
| Behavioral Health | 90836 | Psychotherapy, 45 minutes with patient when performed with an evaluation and management service | 90836: Psychotherapy, 45 minutes with patient when performed with an evaluation and management service |
| Behavioral Health | 90837 | Psychotherapy, 60 minutes with patient | 90837: Psychotherapy, 60 minutes with patient |
| Behavioral Health | 90838 | Psychotherapy, 60 minutes with patient when performed with an evaluation and management service | 90838: Psychotherapy, 60 minutes with patient when performed with an evaluation and management service |
| Behavioral Health | 90846 | Family Psychotherapy without Patient present | 90846: Family Psychotherapy without Patient present |
| Behavioral Health | 90847 | Family Psychotherapy with Patient present | 90847: Family Psychotherapy with Patient present |
| Behavioral Health | 97156 | Family Adaptive Behavior Treatment Guidance. Family adaptive behavior treatment guidance, administered by physician or other qualified healthcare professional (with or without the patient present), faceto-face with guardian(s)/caregiver(s), each 15 minutes | 97156: Family Adaptive Behavior Treatment Guidance. Family adaptive behavior treatment guidance, administered by physician or other qualified healthcare professional (with or without the patient present), faceto-face with guardian(s)/caregiver(s), each 15 minutes |
| Behavioral Health | 99484, 99492-4 | Behavioral health integration services aka Collaborative Care Model (CoCM) | 99484, 99492-4 : Behavioral health integration services aka Collaborative Care Model (CoCM) |
| Behavioral Health | H0046 | Behavioral health-related medical services: verbal interaction, mental health | H0046: Behavioral health-related medical services: verbal interaction, mental health |
| Behavioral Health | H0047 | Behavioral health-related medical services: verbal interaction, substance abuse | H0047: Behavioral health-related medical services: verbal interaction, substance abuse |
| Behavioral Health | H2010 HE | Brief individual medical psychotherapy, mental health | H2010 HE : Brief individual medical psychotherapy, mental health |
| Behavioral Health | H2010 HF | Brief individual medical psychotherapy, substance abuse | H2010 HF: Brief individual medical psychotherapy, substance abuse |
| Behavioral Health | H2015 H2015 HQ | Community Networking - Individual and Group (Innovations and TBI) | H2015 H2015 HQ: Community Networking - Individual and Group (Innovations and TBI) |
| Behavioral Health | H2019 HR | Individual Therapy (Therapeutic behavioral services) | H2019 HR: Individual Therapy (Therapeutic behavioral services) |
| Behavioral Health | H2022 U4 | Transitional Living Skills: The Transitional Living program is designed to aid young adults from age 16 through 21 who are nearing adulthood leaving the foster care, juvenile justice, or mental health systems, in learning the skills needed to succeed independently. Self-sufficiency skills, community reintegration, education, vocational skills and job training/experience are the major focal areas within the program. The proposed program will provide clients with the knowledge and skills necessary to ∙ Maintain stable housing ∙ Maintain productive activity (work or educational program) ∙ Remain free from legal involvement ∙ Become self-sufficient | H2022 U4: Transitional Living Skills: The Transitional Living program is designed to aid young adults from age 16 through 21 who are nearing adulthood leaving the foster care, juvenile justice, or mental health systems, in learning the skills needed to succeed independently. Self-sufficiency skills, community reintegration, education, vocational skills and job training/experience are the major focal areas within the program. The proposed program will provide clients with the knowledge and skills necessary to ∙ Maintain stable housing ∙ Maintain productive activity (work or educational program) ∙ Remain free from legal involvement ∙ Become self-sufficient |
| Behavioral Health | H2023 U4 H2026 U4  HQ for group | Supported Employment (Initial and Maintenance) | H2023 U4 H2026 U4  HQ for group: Supported Employment (Initial and Maintenance) |
| Behavioral Health | H2025 H2025 HQ | Supported Employment - Individual and Group (Innovations and TBI) | H2025 H2025 HQ: Supported Employment - Individual and Group (Innovations and TBI) |
| Behavioral Health | S5110 | Natural Supports Education (Innovations and TBI): Home care training, family; per 15 minutes | S5110: Natural Supports Education (Innovations and TBI): Home care training, family; per 15 minutes |
| Behavioral Health | T1012 U4 | Intensive Recovery Supports: Alcohol and/or substance abuse services, skills development. Established for State Medical Agencies | T1012 U4: Intensive Recovery Supports: Alcohol and/or substance abuse services, skills development. Established for State Medical Agencies |
| Chronic Conditions | 0403T | National Diabetes Prevention Program (NDPP) | 0403T : National Diabetes Prevention Program (NDPP) |
| Chronic Conditions | 99406, 99407 | Smoking and Tobacco Cessation Counseling | 99406, 99407: Smoking and Tobacco Cessation Counseling |
| Chronic Conditions | 99487-90 | Chronic care management services | 99487-90 : Chronic care management services |
| Chronic Conditions | 99495-6 | Transitional care management services | 99495-6 : Transitional care management services |
| Chronic Conditions | G0108 | Diabetes Self Management Education | G0108: Diabetes Self Management Education |
| Dentistry | D9995 | Teledentistry – synchronous; real-time encounter. Reported in addition to other procedures (e.g., diagnostic) delivered to the patient on the date of service. | D9995: Teledentistry – synchronous; real-time encounter. Reported in addition to other procedures (e.g., diagnostic) delivered to the patient on the date of service. |
| Dentistry | D9996 | Teledentistry – asynchronous; information stored and forwarded to dentist for subsequent review Reported in addition to other procedures (e.g., diagnostic) delivered to the patient on the date of service. | D9996: Teledentistry – asynchronous; information stored and forwarded to dentist for subsequent review Reported in addition to other procedures (e.g., diagnostic) delivered to the patient on the date of service. |
| Dentistry | D0140 | Limited oral evaluation – problem focused.  An evaluation limited to a specific oral health problem or complaint. This may require interpretation of information acquired through additional diagnostic procedures. Report additional diagnostic procedures separately. Definitive procedures may be required on the same date as the evaluation. Typically, patients receiving this type of evaluation present with a specific problem and/or dental emergencies, trauma, acute infections, etc. | D0140: Limited oral evaluation – problem focused.  An evaluation limited to a specific oral health problem or complaint. This may require interpretation of information acquired through additional diagnostic procedures. Report additional diagnostic procedures separately. Definitive procedures may be required on the same date as the evaluation. Typically, patients receiving this type of evaluation present with a specific problem and/or dental emergencies, trauma, acute infections, etc. |
| Dentistry | D9430 | Used for live streaming video or telephone with a patient with oral health issues in lieu of an in-person ofce visit. Only allowed for an actual conversation between the member and the provider about oral health issues as their chief complaint. | D9430: Used for live streaming video or telephone with a patient with oral health issues in lieu of an in-person ofce visit. Only allowed for an actual conversation between the member and the provider about oral health issues as their chief complaint. |
| General E&M | 98966 98967 98968 | Initiation of telephone/audio-only visit by patient | 98966 98967 98968: Initiation of telephone/audio-only visit by patient |
| General E&M | 99201 | Office or other outpatient visit for the evaluation and management of a new patient; Straightforward medical decision making. Typically, 10 minutes. | 99201: Office or other outpatient visit for the evaluation and management of a new patient; Straightforward medical decision making. Typically, 10 minutes. |
| General E&M | 99202 | Office or other outpatient visit for the evaluation and management of a new patient; Straightforward medical decision making. Typically, 20 minutes. | 99202: Office or other outpatient visit for the evaluation and management of a new patient; Straightforward medical decision making. Typically, 20 minutes. |
| General E&M | 99203 | Office or other outpatient visit for the evaluation and management of a new patient; Medical decision making of low complexity. Typically, 30 minutes. | 99203: Office or other outpatient visit for the evaluation and management of a new patient; Medical decision making of low complexity. Typically, 30 minutes. |
| General E&M | 99204 | Office or other outpatient visit for the evaluation and management of a new patient; Medical decision making of moderate complexity. Typically, 45 minutes. | 99204: Office or other outpatient visit for the evaluation and management of a new patient; Medical decision making of moderate complexity. Typically, 45 minutes. |
| General E&M | 99205 | Office or other outpatient visit for the evaluation and management of a new patient; Medical decision making of moderate to high complexity. Typically, 60 minutes. | 99205: Office or other outpatient visit for the evaluation and management of a new patient; Medical decision making of moderate to high complexity. Typically, 60 minutes. |
| General E&M | 99211 | Office or other outpatient visit for the evaluation and management of an established patient, that may not require the presence of a physician or other qualified health care professional. Usually, the presenting problem(s) are minimal. Typically, 5 minutes. | 99211: Office or other outpatient visit for the evaluation and management of an established patient, that may not require the presence of a physician or other qualified health care professional. Usually, the presenting problem(s) are minimal. Typically, 5 minutes. |
| General E&M | 99212 | Office or other outpatient visit for the evaluation and management of an established patient; Straightforward medical decision making. Typically, 10 minutes. | 99212: Office or other outpatient visit for the evaluation and management of an established patient; Straightforward medical decision making. Typically, 10 minutes. |
| General E&M | 99213 | Office or other outpatient visit for the evaluation and management of an established patient; Medical decision making of low complexity. Typically, 15 minutes. | 99213: Office or other outpatient visit for the evaluation and management of an established patient; Medical decision making of low complexity. Typically, 15 minutes. |
| General E&M | 99214 | Office or other outpatient visit for the evaluation and management of an established patient; Medical decision making of moderate complexity. Typically, 25 minutes. | 99214: Office or other outpatient visit for the evaluation and management of an established patient; Medical decision making of moderate complexity. Typically, 25 minutes. |
| General E&M | 99215 | Office or other outpatient visit for the evaluation and management of an established patient, which requires at least two of these three key components: a comprehensive history; a comprehensive examination; medical decision making of high complexity. Counseling and/or coordination of care with other physicians, other qualified health care professionals, or agencies are provided consistent with the nature of the problem(s) and the patient's and/or family's needs. Usually, the presenting problem(s) are of moderate to high severity. Typically, 40 minutes are spent face-to-face with the patient and/or family. | 99215: Office or other outpatient visit for the evaluation and management of an established patient, which requires at least two of these three key components: a comprehensive history; a comprehensive examination; medical decision making of high complexity. Counseling and/or coordination of care with other physicians, other qualified health care professionals, or agencies are provided consistent with the nature of the problem(s) and the patient's and/or family's needs. Usually, the presenting problem(s) are of moderate to high severity. Typically, 40 minutes are spent face-to-face with the patient and/or family. |
| General E&M | 99241 | Office consultation for a new or established patient. Usually, the presenting problem(s) are self-limited or minor. Typically, 15 minutes. | 99241: Office consultation for a new or established patient. Usually, the presenting problem(s) are self-limited or minor. Typically, 15 minutes. |
| General E&M | 99242 | Office consultation for a new or established patient; Straightforward medical decision making; Typically, 30 minutes. | 99242: Office consultation for a new or established patient; Straightforward medical decision making; Typically, 30 minutes. |
| General E&M | 99441 | Telephone evaluation and management service by a physician or other qualified health care professional who may report evaluation and management services provided to an established patient, parent, or guardian not originating from a related E/M service provided within the previous 7 days nor leading to an E/M service or procedure within the next 24 hours or soonest available appointment; 5-10 minutes of medical discussion | 99441: Telephone evaluation and management service by a physician or other qualified health care professional who may report evaluation and management services provided to an established patient, parent, or guardian not originating from a related E/M service provided within the previous 7 days nor leading to an E/M service or procedure within the next 24 hours or soonest available appointment; 5-10 minutes of medical discussion |
| General E&M | 99442 | Telephone evaluation and management service by a physician or other qualified health care professional who may report evaluation and management services provided to an established patient, parent, or guardian not originating from a related E/M service provided within the previous 7 days nor leading to an E/M service or procedure within the next 24 hours or soonest available appointment;11-20 minutes of medical discussion | 99442: Telephone evaluation and management service by a physician or other qualified health care professional who may report evaluation and management services provided to an established patient, parent, or guardian not originating from a related E/M service provided within the previous 7 days nor leading to an E/M service or procedure within the next 24 hours or soonest available appointment;11-20 minutes of medical discussion |
| General E&M | 99443 | Telephone evaluation and management service by a physician or other qualified health care professional who may report evaluation and management services provided to an established patient, parent, or guardian not originating from a related E/M service provided within the previous 7 days nor leading to an E/M service or procedure within the next 24 hours or soonest available appointment;21-30 minutes of medical discussion | 99443: Telephone evaluation and management service by a physician or other qualified health care professional who may report evaluation and management services provided to an established patient, parent, or guardian not originating from a related E/M service provided within the previous 7 days nor leading to an E/M service or procedure within the next 24 hours or soonest available appointment;21-30 minutes of medical discussion |
| General E&M | 99451 | Interprofessional telephone/Internet/electronic health record assessment and management service provided by a consultative physician, including a written report to the patient's treating/requesting physician or other qualified health care professional, 5 minutes or more of medical consultative time | 99451: Interprofessional telephone/Internet/electronic health record assessment and management service provided by a consultative physician, including a written report to the patient's treating/requesting physician or other qualified health care professional, 5 minutes or more of medical consultative time |
| General E&M | T1014 | Telehealth transmission, per minute, professional services. | T1014: Telehealth transmission, per minute, professional services. |
| General E&M | 99421 | Online digital evaluation and management service, for an established patient, for up to 7 days, cumulative time during the 7 days; 5–10 minutes | 99421: Online digital evaluation and management service, for an established patient, for up to 7 days, cumulative time during the 7 days; 5–10 minutes |
| General E&M | 99422 | Online digital evaluation and management service, for an established patient, for up to 7 days, cumulative time during the 7 days; 11– 20 minutes | 99422: Online digital evaluation and management service, for an established patient, for up to 7 days, cumulative time during the 7 days; 11– 20 minutes |
| General E&M | 99423 | Online digital evaluation and management service, for an established patient, for up to 7 days, cumulative time during the 7 days; 21 or more minutes | 99423: Online digital evaluation and management service, for an established patient, for up to 7 days, cumulative time during the 7 days; 21 or more minutes |
| General E&M | 99446 | Interprofessional telephone/internet/electronic health records assessment and management service provided by a consultative physician, including a verbal and written report to the patient’s treating/requesting physician or other qualified health care professional; 5-10 minutes of medical consultative discussion and review | 99446: Interprofessional telephone/internet/electronic health records assessment and management service provided by a consultative physician, including a verbal and written report to the patient’s treating/requesting physician or other qualified health care professional; 5-10 minutes of medical consultative discussion and review |
| General E&M | 99447 | Interprofessional telephone/internet/electronic health records assessment and management service provided by a consultative physician, including a verbal and written report to the patient’s treating/requesting physician or other qualified health care professional; 11-20 minutes of medical consultative discussion and review | 99447: Interprofessional telephone/internet/electronic health records assessment and management service provided by a consultative physician, including a verbal and written report to the patient’s treating/requesting physician or other qualified health care professional; 11-20 minutes of medical consultative discussion and review |
| General E&M | 99448 | Interprofessional telephone/internet/electronic health records assessment and management service provided by a consultative physician, including a verbal and written report to the patient’s treating/requesting physician or other qualified health care professional; 21-30 minutes of medical consultative discussion and review | 99448: Interprofessional telephone/internet/electronic health records assessment and management service provided by a consultative physician, including a verbal and written report to the patient’s treating/requesting physician or other qualified health care professional; 21-30 minutes of medical consultative discussion and review |
| General E&M | 99449 | Interprofessional telephone/internet/electronic health records assessment and management service provided by a consultative physician, including a verbal and written report to the patient’s treating/requesting physician or other qualified health care professional; 31 minutes or more of medical consultative discussion and review | 99449: Interprofessional telephone/internet/electronic health records assessment and management service provided by a consultative physician, including a verbal and written report to the patient’s treating/requesting physician or other qualified health care professional; 31 minutes or more of medical consultative discussion and review |
| General E&M | Q3014 | Telehealth originating site facility fee or just “Telehealth facility fee.” Billable by originating site; once per day; same patient, same provider | Q3014: Telehealth originating site facility fee or just “Telehealth facility fee.” Billable by originating site; once per day; same patient, same provider |
| Home visit | 99347 | Home visit for the evaluation and management of an established patient, which requires at least two of these three key components: A problem focused interval history; A problem focused examination; and Straightforward medical decision making. Presenting problem(s) are self-limited or minor. Typically, 15 minutes are spent face-to-face with the patient/family. | 99347: Home visit for the evaluation and management of an established patient, which requires at least two of these three key components: A problem focused interval history; A problem focused examination; and Straightforward medical decision making. Presenting problem(s) are self-limited or minor. Typically, 15 minutes are spent face-to-face with the patient/family. |
| Home visit | 99348 | Home visit for the evaluation and management of an established patient, which requires at least two of these three key components: An expanded problem focused interval history; An expanded problem focused examination; and Medical decision making of low complexity. Presenting problem(s) are low to moderate severity. Typically, 25 minutes are spent face-to-face with the patient/family. | 99348: Home visit for the evaluation and management of an established patient, which requires at least two of these three key components: An expanded problem focused interval history; An expanded problem focused examination; and Medical decision making of low complexity. Presenting problem(s) are low to moderate severity. Typically, 25 minutes are spent face-to-face with the patient/family. |
| Home visit | 99349 | Home visit for the evaluation and management of an established patient, which requires at least two of these three key components: A detailed interval history; A detailed examination; and Medical decision making of moderate complexity. Presenting problem(s) are low to moderate to high severity. Typically, 40 minutes are spent face-to-face with the patient/family. | 99349: Home visit for the evaluation and management of an established patient, which requires at least two of these three key components: A detailed interval history; A detailed examination; and Medical decision making of moderate complexity. Presenting problem(s) are low to moderate to high severity. Typically, 40 minutes are spent face-to-face with the patient/family. |
| Home visit | 99350 | Home visit for the evaluation and management of an established patient, which requires at least two of these three key components: A comprehensive interval history; A comprehensive examination; and Medical decision making of moderate to high complexity. Presenting problem(s) are low to moderate to high severity. The patient may be unstable or may have developed a significant new problem requiring immediate physician attention. Typically, 60 minutes are spent face-to-face with the patient/family. | 99350: Home visit for the evaluation and management of an established patient, which requires at least two of these three key components: A comprehensive interval history; A comprehensive examination; and Medical decision making of moderate to high complexity. Presenting problem(s) are low to moderate to high severity. The patient may be unstable or may have developed a significant new problem requiring immediate physician attention. Typically, 60 minutes are spent face-to-face with the patient/family. |
| Individuals with development disabilities (IDD) or Cognitive Impairment | T1024 GN | Initial (or Follow Up) Interdisciplinary Psychosocial and Developmental Evaluation rendered by a Speech Therapist | T1024 GN: Initial (or Follow Up) Interdisciplinary Psychosocial and Developmental Evaluation rendered by a Speech Therapist |
| Individuals with development disabilities (IDD) or Cognitive Impairment | T1024 GO | Initial (or Follow Up) Interdisciplinary Psychosocial and Developmental Evaluation rendered by an Occupational Therapist | T1024 GO: Initial (or Follow Up) Interdisciplinary Psychosocial and Developmental Evaluation rendered by an Occupational Therapist |
| Individuals with development disabilities (IDD) or Cognitive Impairment | T1024 GP | Initial (or Follow Up) Interdisciplinary Psychosocial and Developmental Evaluation rendered by a Physical Therapist | T1024 GP: Initial (or Follow Up) Interdisciplinary Psychosocial and Developmental Evaluation rendered by a Physical Therapist |
| Individuals with development disabilities (IDD) or Cognitive Impairment | T1024 HN | Initial (or Follow Up) Interdisciplinary Psychosocial and Developmental Evaluation rendered by an ITDS | T1024 HN: Initial (or Follow Up) Interdisciplinary Psychosocial and Developmental Evaluation rendered by an ITDS |
| Individuals with development disabilities (IDD) or Cognitive Impairment | T1024 TL | Initial (or Follow Up) Interdisciplinary Psychosocial and Developmental Evaluation rendered by a Licensed Early Intervention Professional | T1024 TL: Initial (or Follow Up) Interdisciplinary Psychosocial and Developmental Evaluation rendered by a Licensed Early Intervention Professional |
| Individuals with development disabilities (IDD) or Cognitive Impairment | T2025 | Specialized Consultative Services (Innovations and TBI): Waiver services; not otherwise specified (nos) | T2025: Specialized Consultative Services (Innovations and TBI): Waiver services; not otherwise specified (nos) |
| Individuals with development disabilities (IDD) or Cognitive Impairment | T2033 T2033 HI T2033 TF | Supported Living – Level 1, Level 2, Level 3 (Innovations) | T2033 T2033 HI T2033 TF: Supported Living – Level 1, Level 2, Level 3 (Innovations) |
| Interim Perinatal Care Guidance | S0280 | Providers shall bill this code after the pregnancy risk screening tool has been completed. Medical home program, comprehensive care coordination and planning, initial plan. Note: Must be billed by the practice | S0280: Providers shall bill this code after the pregnancy risk screening tool has been completed. Medical home program, comprehensive care coordination and planning, initial plan. Note: Must be billed by the practice |
| Maternal and Perinatal Health | 99501 | A home visit for postnatal assessment and follow-up care is designed to deliver health, social support, and/or educational services directly to families in their homes. A home visit for postnatal assessment and follow-up care is a means to follow up on the mother’s health; to counsel on family planning and infant care; and to arrange for additional appointments for the infant and mother. Eligible provider: must be rendered by a registered nurse. | 99501: A home visit for postnatal assessment and follow-up care is designed to deliver health, social support, and/or educational services directly to families in their homes. A home visit for postnatal assessment and follow-up care is a means to follow up on the mother’s health; to counsel on family planning and infant care; and to arrange for additional appointments for the infant and mother. Eligible provider: must be rendered by a registered nurse. |
| Maternal and Perinatal Health | 99502 | A home visit for newborn care and assessment delivers health, social support, and/or educational services directly to families in their homes. A home visit for newborn care and assessment is a means to follow up on the infant’s health; to counsel on infant care; to follow up on newborn screening; and to arrange for additional appointments for the infant. Eligible provider: must be rendered by a registered nurse. | 99502: A home visit for newborn care and assessment delivers health, social support, and/or educational services directly to families in their homes. A home visit for newborn care and assessment is a means to follow up on the infant’s health; to counsel on infant care; to follow up on newborn screening; and to arrange for additional appointments for the infant. Eligible provider: must be rendered by a registered nurse. |
| Maternal and Perinatal Health | 96156, 8, 9 | Medical Lactation Support | 34426520: Medical Lactation Support |
| Maternal and Perinatal Health | S9442 | Childbirth education is a series of classes designed to help pregnant women and their support person to understand the changes experienced during pregnancy, to prepare for the labor and delivery experience, and to understand the postpartum period, including, but not limited to, the importance of proper postpartum care for the mother and the child. Eligible provider: must be rendered by a certified childbirth educator who is eligible to bill for this service. | S9442: Childbirth education is a series of classes designed to help pregnant women and their support person to understand the changes experienced during pregnancy, to prepare for the labor and delivery experience, and to understand the postpartum period, including, but not limited to, the importance of proper postpartum care for the mother and the child. Eligible provider: must be rendered by a certified childbirth educator who is eligible to bill for this service. |
| Nutrition | 97802, 97803 | Dietary Evaluation and Counseling: Medical nutrition therapy; initial assessment and intervention, individual, face-to-face with the patient, each 15 minutes. | 97802, 97803: Dietary Evaluation and Counseling: Medical nutrition therapy; initial assessment and intervention, individual, face-to-face with the patient, each 15 minutes. |
| Occupational Therapy | 97165 | Occupational therapy evaluation, low complexity; typically, 30 minutes are spent face-to-face with patient and/or family. | 97165: Occupational therapy evaluation, low complexity; typically, 30 minutes are spent face-to-face with patient and/or family. |
| Occupational Therapy | 97166 | Occupational therapy evaluation, moderate complexity, requiring these components; typically, 45 minutes are spent face-to-face with patient and/or family. | 97166: Occupational therapy evaluation, moderate complexity, requiring these components; typically, 45 minutes are spent face-to-face with patient and/or family. |
| Occupational Therapy | 97167 | Occupational therapy evaluation, high complexity, requiring these components; typically, 60 minutes are spent face-to-face with patient and/or family. | 97167: Occupational therapy evaluation, high complexity, requiring these components; typically, 60 minutes are spent face-to-face with patient and/or family. |
| Occupational Therapy | 97168 | Re-evaluation of occupational therapy established plan of care; typically, 30 minutes are spent face-to-face with the patient and/or family. | 97168: Re-evaluation of occupational therapy established plan of care; typically, 30 minutes are spent face-to-face with the patient and/or family. |
| Occupational Therapy | 97533 | Sensory integrative techniques to enhance sensory processing and promote adaptive responses to environmental demands, direct (one-on-one) patient contact, each 15 minutes | 97533: Sensory integrative techniques to enhance sensory processing and promote adaptive responses to environmental demands, direct (one-on-one) patient contact, each 15 minutes |
| Occupational Therapy | 92526 | Treatment of swallowing dysfunction and/or oral function for feeding | 92526: Treatment of swallowing dysfunction and/or oral function for feeding |
| Pediatrics | 96110 | Developmental screening (e.g. developmental milestone survey, speech and language delay screen), with scoring and documentation, per standardized instrument | 96110: Developmental screening (e.g. developmental milestone survey, speech and language delay screen), with scoring and documentation, per standardized instrument |
| Pediatrics | 96111 | Developmental Testing, extended | 96111: Developmental Testing, extended |
| Pediatrics | 96127 | Brief emotional/behavioral assessment (e.g. depression inventory, attention-deficit/hyperactivity disorder (ADHD) scale), with scoring and documentation, per standardized instrument | 96127: Brief emotional/behavioral assessment (e.g. depression inventory, attention-deficit/hyperactivity disorder (ADHD) scale), with scoring and documentation, per standardized instrument |
| Pediatrics | 96160 | Administration of patient-focused health risk assessment instrument (e.g. health hazard appraisal) with scoring and documentation, per standardized instrument | 96160: Administration of patient-focused health risk assessment instrument (e.g. health hazard appraisal) with scoring and documentation, per standardized instrument |
| Pediatrics | 96161 | Administration of caregiver-focused health risk assessment instrument (e.g. depression inventory) for the benefit of the patient, with scoring and documentation, per standardized instrument Note: Maternal depression screens may be billed to the child’s Medicaid as CPT 96161. NC Medicaid will reimburse providers for up to 4 maternal depression risk screens administered to mothers during the infant’s first year postpartum. | 96161: Administration of caregiver-focused health risk assessment instrument (e.g. depression inventory) for the benefit of the patient, with scoring and documentation, per standardized instrument Note: Maternal depression screens may be billed to the child’s Medicaid as CPT 96161. NC Medicaid will reimburse providers for up to 4 maternal depression risk screens administered to mothers during the infant’s first year postpartum. |
| Pediatrics | 97151 | Behavior identification assessment, administered by a physician or other qualified healthcare professional, each 15 minutes of the physician’s or other qualified healthcare professional’s time face-to-face with patient and/or guardian(s)/caregiver(s) administering assessments and discussing findings and recommendations, and nonface-to-face analyzing past data, scoring/interpreting the assessment, and preparing the report/treatment plan. | 97151: Behavior identification assessment, administered by a physician or other qualified healthcare professional, each 15 minutes of the physician’s or other qualified healthcare professional’s time face-to-face with patient and/or guardian(s)/caregiver(s) administering assessments and discussing findings and recommendations, and nonface-to-face analyzing past data, scoring/interpreting the assessment, and preparing the report/treatment plan. |
| Pediatrics | 97152 | Behavior identification supporting assessment, administered by one technician under the direction of a physician or other qualified healthcare professional, faceto-face with the patient, each 15 minute | 97152: Behavior identification supporting assessment, administered by one technician under the direction of a physician or other qualified healthcare professional, faceto-face with the patient, each 15 minute |
| Pediatrics | 97153 | Adaptive behavior treatment by protocol, administered by technician under the direction of a physician or other qualified healthcare professional, face-to-face with one patient, each 15 minutes. | 97153: Adaptive behavior treatment by protocol, administered by technician under the direction of a physician or other qualified healthcare professional, face-to-face with one patient, each 15 minutes. |
| Pediatrics | 97154 | Group adaptive behavior treatment by protocol, administered by technician under the direction of a physician or other qualified healthcare professional, faceto-face with two or more patients, each 15 minutes. | 97154: Group adaptive behavior treatment by protocol, administered by technician under the direction of a physician or other qualified healthcare professional, faceto-face with two or more patients, each 15 minutes. |
| Pediatrics | 97155 | Adaptive behavior treatment with protocol modification, administered by physician or other qualified healthcare professional, which may include simultaneous direction of technician, face-to-face with one patient, each 15 minutes. | 97155: Adaptive behavior treatment with protocol modification, administered by physician or other qualified healthcare professional, which may include simultaneous direction of technician, face-to-face with one patient, each 15 minutes. |
| Pediatrics | 97157 | Multiple-family group adaptive behavior treatment guidance, administered by physician or other qualified healthcare professional (without the patient present), face-to-face with multiple sets of guardians/caregivers, each 15 minutes. | 97157: Multiple-family group adaptive behavior treatment guidance, administered by physician or other qualified healthcare professional (without the patient present), face-to-face with multiple sets of guardians/caregivers, each 15 minutes. |
| Pediatrics | 97158 | Group adaptive behavior treatment with protocol modification, administered by physician or other qualified healthcare professional, face-to-face with multiple patients, each 15 minutes. | 97158: Group adaptive behavior treatment with protocol modification, administered by physician or other qualified healthcare professional, face-to-face with multiple patients, each 15 minutes. |
| Pediatrics | 99381 | Initial comprehensive preventive medicine evaluation and management of an individual including an age and gender appropriate history, examination, counseling/anticipatory guidance/risk factor reduction interventions, and the ordering of laboratory/diagnostic procedures, new patient, early childhood (younger than 1 year) | 99381: Initial comprehensive preventive medicine evaluation and management of an individual including an age and gender appropriate history, examination, counseling/anticipatory guidance/risk factor reduction interventions, and the ordering of laboratory/diagnostic procedures, new patient, early childhood (younger than 1 year) |
| Pediatrics | 99382 | Initial comprehensive preventative medicine evaluation and management of an individual including an age and gender appropriate history, examination, counseling/anticipatory guidance/risk factor reduction interventions, and the ordering of laboratory/diagnostic procedures, new patient, early childhood (age 1 through 4 years) | 99382: Initial comprehensive preventative medicine evaluation and management of an individual including an age and gender appropriate history, examination, counseling/anticipatory guidance/risk factor reduction interventions, and the ordering of laboratory/diagnostic procedures, new patient, early childhood (age 1 through 4 years) |
| Pediatrics | 99383 | Initial comprehensive preventative medicine evaluation and management of an individual including an age and gender appropriate history, examination, counseling/anticipatory guidance/risk factor reduction interventions, and the ordering of laboratory/diagnostic procedures, new patient, late childhood (age 5 through 11 years) | 99383: Initial comprehensive preventative medicine evaluation and management of an individual including an age and gender appropriate history, examination, counseling/anticipatory guidance/risk factor reduction interventions, and the ordering of laboratory/diagnostic procedures, new patient, late childhood (age 5 through 11 years) |
| Pediatrics | 99384 | Initial comprehensive preventative medicine evaluation and management of an individual including an age and gender appropriate history, examination, counseling/anticipatory guidance/risk factor reduction interventions, and the ordering of laboratory/diagnostic procedures, new patient, adolescent (age 12 through 17 years) | 99384: Initial comprehensive preventative medicine evaluation and management of an individual including an age and gender appropriate history, examination, counseling/anticipatory guidance/risk factor reduction interventions, and the ordering of laboratory/diagnostic procedures, new patient, adolescent (age 12 through 17 years) |
| Pediatrics | 99385 | Initial comprehensive preventative medicine evaluation and management of an individual including an age and gender appropriate history, examination, counseling/anticipatory guidance/risk factor reduction interventions, and the ordering of laboratory/diagnostic procedures, new patient, 18 through 39 years | 99385: Initial comprehensive preventative medicine evaluation and management of an individual including an age and gender appropriate history, examination, counseling/anticipatory guidance/risk factor reduction interventions, and the ordering of laboratory/diagnostic procedures, new patient, 18 through 39 years |
| Pediatrics | 99391 | Periodic comprehensive preventive medicine reevaluation and management of an individual including an age and gender appropriate history, examination, counseling/anticipatory guidance/risk factor reduction interventions, and the ordering of laboratory/diagnostic procedures, established patient, infant (younger than 1 year) | 99391: Periodic comprehensive preventive medicine reevaluation and management of an individual including an age and gender appropriate history, examination, counseling/anticipatory guidance/risk factor reduction interventions, and the ordering of laboratory/diagnostic procedures, established patient, infant (younger than 1 year) |
| Pediatrics | 99392 | Periodic comprehensive preventative medicine reevaluation and management of an individual including an age and gender appropriate history, examination, counseling/anticipatory guidance/risk factor reduction interventions, and the ordering of laboratory/diagnostic procedures, established patient, early childhood (1 through 4 years) | 99392: Periodic comprehensive preventative medicine reevaluation and management of an individual including an age and gender appropriate history, examination, counseling/anticipatory guidance/risk factor reduction interventions, and the ordering of laboratory/diagnostic procedures, established patient, early childhood (1 through 4 years) |
| Pediatrics | 99393 | Periodic comprehensive preventative medicine reevaluation and management of an individual including an age and gender appropriate history, examination, counseling/anticipatory guidance/risk factor reduction interventions, and the ordering of laboratory/diagnostic procedures, established patient, late childhood (5 through 11 years) | 99393: Periodic comprehensive preventative medicine reevaluation and management of an individual including an age and gender appropriate history, examination, counseling/anticipatory guidance/risk factor reduction interventions, and the ordering of laboratory/diagnostic procedures, established patient, late childhood (5 through 11 years) |
| Pediatrics | 99394 | Periodic comprehensive preventative medicine reevaluation and management of an individual including an age and gender appropriate history, examination, counseling/anticipatory guidance/risk factor reduction interventions, and the ordering of laboratory/diagnostic procedures, established patient, adolescent (12 through 17 years) | 99394: Periodic comprehensive preventative medicine reevaluation and management of an individual including an age and gender appropriate history, examination, counseling/anticipatory guidance/risk factor reduction interventions, and the ordering of laboratory/diagnostic procedures, established patient, adolescent (12 through 17 years) |
| Pediatrics | 99395 | Periodic comprehensive preventative medicine reevaluation and management of an individual including an age and gender appropriate history, examination, counseling/anticipatory guidance/risk factor reduction interventions, and the ordering of laboratory/diagnostic procedures, established patient, 18 through 39 years | 99395: Periodic comprehensive preventative medicine reevaluation and management of an individual including an age and gender appropriate history, examination, counseling/anticipatory guidance/risk factor reduction interventions, and the ordering of laboratory/diagnostic procedures, established patient, 18 through 39 years |
| Pediatrics | 92506 | Evaluation and management of speech, language, voice, communication, and/or auditory processing | 92506: Evaluation and management of speech, language, voice, communication, and/or auditory processing |
| Peer | 98960-2 | Self-management education & training, face-to-face | 98960-2 : Self-management education & training, face-to-face |
| Peer | H0038 | Self-help/peer services | H0038 : Self-help/peer services |
| Personal care and habilitation | T1027 | Family training & counseling | T1027: Family training & counseling |
| Personal care and habilitation | T2013 T2013 HQ | Life Skills Training - Individual and Group (TBI) | T2013 T2013 HQ: Life Skills Training - Individual and Group (TBI) |
| Personal care and habilitation | T2041 | Resource Facilitation (TBI): Supports brokerage, self-directed, waiver; per 15 minutes | T2041: Resource Facilitation (TBI): Supports brokerage, self-directed, waiver; per 15 minutes |
| Personal care and habilitation | YA389 | Long-Term Vocational Support Services (Extended Services-IDD) | YA389: Long-Term Vocational Support Services (Extended Services-IDD) |
| Personal care and habilitation | YM390 - Ind YP640 - Group | Supported Employment-IDD | YM390 - Ind YP640 - Group: Supported Employment-IDD |
| Physical Therapy | 97161 | Physical therapy evaluation: low complexity, typically, 20 minutes are spent face-to-face with the patient and/or family. | 97161: Physical therapy evaluation: low complexity, typically, 20 minutes are spent face-to-face with the patient and/or family. |
| Physical Therapy | 97162 | Physical therapy evaluation: moderate complexity; typically, 30 minutes are spent face-to-face with the patient and/or family. | 97162: Physical therapy evaluation: moderate complexity; typically, 30 minutes are spent face-to-face with the patient and/or family. |
| Physical Therapy | 97163 | Physical therapy evaluation; high complexity; typically, 45 minutes are spent face-to-face with the patient and/or family. | 97163: Physical therapy evaluation; high complexity; typically, 45 minutes are spent face-to-face with the patient and/or family. |
| Physical Therapy | 97164 | Re-evaluation of physical therapy established plan of care; typically, 20 minutes are spent face-to-face with the patient and/or family. | 97164: Re-evaluation of physical therapy established plan of care; typically, 20 minutes are spent face-to-face with the patient and/or family. |
| Remote Monitoring | 99453 | Remote monitoring of physiologic parameter(s)(e.g., weight, blood pressure, pulse oximetry, respiratory flow rate), initial; set-up and patient education on use of equipment | 99453: Remote monitoring of physiologic parameter(s)(e.g., weight, blood pressure, pulse oximetry, respiratory flow rate), initial; set-up and patient education on use of equipment |
| Remote Monitoring | 99454 | Remote monitoring of physiologic parameter(s)(e.g., weight, blood pressure, pulse oximetry, respiratory flow rate), initial; device(s) supply with daily recording(s) or programmed alert(s) transmission | 99454: Remote monitoring of physiologic parameter(s)(e.g., weight, blood pressure, pulse oximetry, respiratory flow rate), initial; device(s) supply with daily recording(s) or programmed alert(s) transmission |
| Remote Monitoring | 99457 | Remote physiologic monitoring treatment management services, clinical staff/physician/other qualified health care professional time in a calendar month requiring interactive communication with the patient/caregiver during the month; first 20 minutes | 99457: Remote physiologic monitoring treatment management services, clinical staff/physician/other qualified health care professional time in a calendar month requiring interactive communication with the patient/caregiver during the month; first 20 minutes |
| Remote Monitoring | 99458 | Remote physiologic monitoring treatment management services, clinical staff/physician/other qualified health care professional time in a calendar month requiring interactive communication with the patient/caregiver during the month; each additional 20 minutes | 99458: Remote physiologic monitoring treatment management services, clinical staff/physician/other qualified health care professional time in a calendar month requiring interactive communication with the patient/caregiver during the month; each additional 20 minutes |
| Remote Monitoring | 99473 | Self-measured blood pressure using a device validated for clinical accuracy; patient education/training and device calibration. | 99473: Self-measured blood pressure using a device validated for clinical accuracy; patient education/training and device calibration. |
| Remote Monitoring | 99474 | Separate self-measurements of two readings one minute apart, twice daily over a 30-day period (minimum of 12 readings), collection of data reported by the patient and/or caregiver to the physician or other qualified health professional, with report of average systolic and diastolic pressures and subsequent communication of a treatment plan to the patient. | 99474: Separate self-measurements of two readings one minute apart, twice daily over a 30-day period (minimum of 12 readings), collection of data reported by the patient and/or caregiver to the physician or other qualified health professional, with report of average systolic and diastolic pressures and subsequent communication of a treatment plan to the patient. |
| Remote Monitoring | 99091 | Collection and interpretation of physiologic data (e.g. ECG, blood pressure, glucose monitoring) digitally stored and/or transmitted by the patient and/or caregiver to the physician or other qualified healthcare professional, qualified by education, training, licensure/regulation (when applicable), requiring a minimum of 30 minutes of time, each 30 days.” | 99091: Collection and interpretation of physiologic data (e.g. ECG, blood pressure, glucose monitoring) digitally stored and/or transmitted by the patient and/or caregiver to the physician or other qualified healthcare professional, qualified by education, training, licensure/regulation (when applicable), requiring a minimum of 30 minutes of time, each 30 days.” |
| Remote Monitoring | G2012 | Brief communication technology-based service, e.g., virtual check-in, by a physician or other qualified health care professional who can report evaluation and management services, provided to an established patient, not originating from a related E/M service provided within the previous 7 days nor leading to an E/M service or procedure within the next 24 hours or soonest available appointment; 5-10 minutes of medical discussion. G2012 can be billed when the virtual communication occurred via a telephone call | G2012: Brief communication technology-based service, e.g., virtual check-in, by a physician or other qualified health care professional who can report evaluation and management services, provided to an established patient, not originating from a related E/M service provided within the previous 7 days nor leading to an E/M service or procedure within the next 24 hours or soonest available appointment; 5-10 minutes of medical discussion. G2012 can be billed when the virtual communication occurred via a telephone call |
| Remote Monitoring | G2064-5 | Principal care management services | G2064-5 : Principal care management services |
| Remote Patient Monitoring | G2010 | Remote evaluation of recorded video and/or images submitted by an established patient (e.g., store and forward), including interpretation with follow-up with the patient within 24 hours, not originating from a related evaluation and management (E/M) service provided within the previous 7 days nor leading to an E/M service or procedure within the next 24 hours or soonest available appointment. | G2010: Remote evaluation of recorded video and/or images submitted by an established patient (e.g., store and forward), including interpretation with follow-up with the patient within 24 hours, not originating from a related evaluation and management (E/M) service provided within the previous 7 days nor leading to an E/M service or procedure within the next 24 hours or soonest available appointment. |
| Respiratory | 94664 | Demonstration and/or evaluation of patient utilization of an aerosol generator nebulizer, metered dose inhaler or IPPB device | 94664: Demonstration and/or evaluation of patient utilization of an aerosol generator nebulizer, metered dose inhaler or IPPB device |
| Speech Therapy | 92507 | Speech/Hearing Therapy | 92507: Speech/Hearing Therapy |
| Speech Therapy | 92508 | Speech/Hearing Therapy | 92508: Speech/Hearing Therapy |
| Speech Therapy | 92521 | Evaluation of Speech Fluency | 92521: Evaluation of Speech Fluency |
| Speech Therapy | 92522 | Evaluate Speech Production | 92522: Evaluate Speech Production |
| Speech Therapy | 92523 | Speech Sound Lang Comprehension | 92523: Speech Sound Lang Comprehension |
| Speech Therapy | 92524 | Behavioral and qualitative analysis of voice and resonance | 92524: Behavioral and qualitative analysis of voice and resonance |
| Speech Therapy | 92607 | Evaluation for prescription for speech-generating augmentative and alternative communication device, face-to-face with the patient; first hour | 92607: Evaluation for prescription for speech-generating augmentative and alternative communication device, face-to-face with the patient; first hour |
| Speech Therapy | 92608 | Evaluation for prescription for speech-generating augmentative and alternative communication device, face-to-face with the patient; each additional 30 minutes | 92608: Evaluation for prescription for speech-generating augmentative and alternative communication device, face-to-face with the patient; each additional 30 minutes |
| Speech Therapy | 97129 | Therapeutic interventions that focus on cognitive function (e.g., attention, memory, reasoning, executive functioning, problem solving and/or pragmatic functioning) and compensatory strategies to manage the performance of an activity (e.g., managing time or schedules, initiating, organizing, and sequencing tasks), direct (one-to-one) patient contact; initial 15 minutes | 97129: Therapeutic interventions that focus on cognitive function (e.g., attention, memory, reasoning, executive functioning, problem solving and/or pragmatic functioning) and compensatory strategies to manage the performance of an activity (e.g., managing time or schedules, initiating, organizing, and sequencing tasks), direct (one-to-one) patient contact; initial 15 minutes |
| Speech Therapy | 97130 | Therapeutic interventions that focus on cognitive function & compensatory strategies, direct patient contact. Additional 15 mins (follow up to 97129) | 97130: Therapeutic interventions that focus on cognitive function & compensatory strategies, direct patient contact. Additional 15 mins (follow up to 97129) |
| Speech Therapy | 97532 | Cognitive Rehabilitation (TBI): cognitive skills development to improve attention, memory and problem-solving, with direct one-on-one patient contact by the qualified professional, each 15 minutes. | 97532: Cognitive Rehabilitation (TBI): cognitive skills development to improve attention, memory and problem-solving, with direct one-on-one patient contact by the qualified professional, each 15 minutes. |
| Speech Therapy | S9152 | Speech Therapy, Re-Eval | S9152: Speech Therapy, Re-Eval |
| Maternal and Perinatal Health | 59410 | Vaginal delivery only (with or without episiotomy and/or forceps); including postpartum care | 59410: Vaginal delivery only (with or without episiotomy and/or forceps); including postpartum care |
| Maternal and Perinatal Health | 59515 | Cesarean delivery only; including postpartum care | 59515: Cesarean delivery only; including postpartum care |
| Pediatrics | 90460 | Immunization administration through 18 years of age via any route of administration, with counseling by physician or other qualified health care professional; first or only component of each vaccine or toxoid administered Note: Providers should code when the vaccine is administered. Vaccine counseling may occur via telemedicine at any time prior to the administration of the vaccine. If vaccine counseling is provided in-person, the 90460 code should be billed as usual. If vaccine counseling as described by this code is not provided, use the appropriate vaccine administration code(s). | 90460: Immunization administration through 18 years of age via any route of administration, with counseling by physician or other qualified health care professional; first or only component of each vaccine or toxoid administered Note: Providers should code when the vaccine is administered. Vaccine counseling may occur via telemedicine at any time prior to the administration of the vaccine. If vaccine counseling is provided in-person, the 90460 code should be billed as usual. If vaccine counseling as described by this code is not provided, use the appropriate vaccine administration code(s). |
